# Flavor and Other Quality Traits of Tomato Cultivars Bred for Diverse Production Systems as Revealed in Organic Low-Input Management

**DOI:** 10.3389/fnut.2022.916642

**Published:** 2022-07-14

**Authors:** Cut Erika, Detlef Ulrich, Marcel Naumann, Inga Smit, Bernd Horneburg, Elke Pawelzik

**Affiliations:** ^1^Division Quality of Plant Products, Department of Crop Sciences, University of Göttingen, Göttingen, Germany; ^2^Institute for Ecological Chemistry, Plant Analysis and Stored Product Protection, Julius Kühn-Institute (JKI), Federal Research Centre for Cultivated Plants, Quedlinburg, Germany; ^3^Section of Genetic Resources and Organic Plant Breeding, Department of Crop Sciences, University of Göttingen, Göttingen, Germany

**Keywords:** tomato cultivars, VOC, organic low-input production, flavor, breeders’ sensory test, phenylethyl alcohol, 2-isobutylthiazole

## Abstract

This study was conducted to determine the volatile organic compounds (VOCs) associated with fruit flavor in diverse tomato cultivars (salad and cocktail cultivars) under organic low-input production. For this objective, 60 cultivars deriving from very diverse breeding programs 1880–2015 were evaluated in 2015, and a subset of 20 cultivars was selected for further evaluation in 2016. The diversity of instrumentally determined traits, especially for VOCs concentration and sensory properties (fruit firmness, juiciness, skin firmness, sweetness, sourness, aroma, and acceptability), was investigated at two harvest dates. The evaluation of the cultivars exhibited a wide range of variation for all studied traits, with the exception of a few VOCs. Cultivar had the most important effect on all instrumentally determined traits, while the influence of cultivar × harvest date × year interaction was significant for 17 VOCs, but not for total soluble solid (TSS) and titratable acidity (TA). The VOCs with the highest proportion (>8%) were hexanal, 6-methyl-5-heptene-2-one, 2-isobutylthiazole, and (*E*)-2-hexenal, which were identified in all cultivars. Twelve VOCs significantly correlated with one or more sensory attributes and these VOCs also allowed differentiation of the fruit type. Among these VOCs, phenylethyl alcohol and benzyl alcohol positively correlated with acceptability in the cocktail cultivars, whereas 2-isobuthylthiazole and 6-methyl-5-hepten-2-ol negatively correlated with acceptability in the salad cultivars. As a result of this study, organic breeders are recommended to use cultivars from a wide range of breeding programs to improve important quality and agronomic traits. As examples, salad tomatoes “Campari F_1_”, “Green Zebra”, and “Auriga”, as well as cocktail tomatoes “Supersweet 100 F_1_”, “Sakura F_1_”, and “Black Cherry” showed higher scores for the sensory attributes aroma and acceptability under organic low-input growing conditions. It remains a challenge for breeders and growers to reduce the trade-off of yield and quality.

## Introduction

Tomato (*Solanum lycopersicum* L.) is currently one of the most important vegetable crops in the world (1), with consumption of fresh tomatoes and products at 20.2 kg capita^–1^ year^–1^ in 2019 (2). The tomato fruit constitutes an essential component of the human diet as it is a source of minerals, vitamins, and phytochemicals (3). For decades, domestication and the considerable attempts in tomato breeding have mainly focused on improving agronomic traits, such as fruit yield and weight, and, to a lesser extent, color and shape, firmness, disease resistance, and adaptation to different growing environments (4–6). While these approaches have significantly increased productivity, they have also shown negative impacts on the sensory and nutritional quality of commercial modern cultivars, which are often perceived by consumers as having less flavor (1, 7, 8). In recent years, the consumer demand for tomatoes with better flavor has increased, which is accompanied by ongoing breeding attempts toward the production of genotypes with improved sensory qualities and with multidisciplinary approaches to sensory assessment of the fruits (9, 10).

The production system can have a greater influence on fruit quality, including the concentration of bioactive compounds, such as phenols and lycopene, than on fruit yield, and the contents of these compounds are often higher in fruits from organic and low-input systems than in fruits from conventional production (11). Field-grown tomatoes have been reported to have higher levels of VOCs than greenhouse-grown fruits (12), but some cultivars e.g., Campari F_1_, have been developed for superior flavor in protected cropping. Flavor properties are important breeding targets to meet consumer demands for tomatoes (13, 14). In addition, the increasing interest in environmentally friendly produced food (15) may encourage breeders to develop cultivars with excellent flavor characteristics for organic low-input cultivation as well. Flavor of a fresh fruit is the sum of an interaction between taste and olfaction that results from a complex interaction of sugars (glucose, fructose), organic acids (citrate, malate, ascorbate, glutamate), and volatile compounds (VOCs) (16, 17). Sugars and acids activate taste receptors, while various VOCs stimulate olfactory receptors (18, 19). Aroma is a very complex trait (20), and its perception is not the result of the action of a single VOC but the result of interactions between different VOCs (16). Texture is also connected to the formation of VOCs, as it is also related to the degradation of cell walls and membranes. At the basic level, cell wall disruption stimulates contact between enzymes and substrates involved in the release of VOCs (21). In addition to many primary and secondary metabolites, such as sugars, amino acids, fatty acids, and carotenoids that can directly influence the sensory properties of the fruit, they are also precursors of some important VOCs of tomato (22). A combination of taste, aroma, and mouth-feel characteristics had major contribution to its flavor. The mechanisms behind flavor characteristics and sensory variations in tomato fruit have been studied to a limited extent (23). Daoud et al. (24) found a good correlation of the sensory attribute aroma with the general taste perception and with texture and, also, with the instrumentally determined sum parameter VOCs. Texture perception is the sensation perceived when eating, and the greatest contributor to texture of tomato products is insoluble solids, which account for approximately 10–20% of the total solids in the cell wall of the fruit (25, 26).

The characteristic sweet–sour flavor of tomato fruit is not solely the result of the interactions of the non-volatile compounds (i.e., sugars, acids, and amino acids) in the fruit (22), but it is determined by a complex combination of volatile and nonvolatile metabolites, which is not yet well understood (1). In strawberry, flavor has apparently been declared an important breeding target after yield and shelf life, to which special attention is paid (27), while tomato has been described as an excellent and important model organism for studies on fleshy fruit to investigate flavor at the molecular level (7, 28). Previous sensory studies on tomato have shown that flavor is the most important characteristic to improve the sensory quality of fruits and is necessary to meet consumers’ expectations (19, 25, 29).

While studies on the chemistry and variability of quality parameters of tomatoes, especially their flavor, compounds are widely available (30, 31); knowledge about chemical properties and VOC accumulation in tomatoes grown in organic low-input conditions and their correlation with general and specific sensory attributes is scarce. In this respect, the characterization of flavor-associated traits relevant to sensory properties is of interest, as they are increasingly valued by consumers.

This study is based on field trials under organic low-input conditions, in which 60 salad and cocktail tomato cultivars were cultivated in 2015, and a subset of 20 cultivars was grown in 2016. First results have been recently published by Chea et al. (32), focusing on identifying cultivars with better growth, yield, and selected fruit quality traits. The aim of the present study was to characterize the cultivars with respect to the variation of their sensory quality under low-input conditions, with the focus on the instrumentally determined traits and on the sensory quality, as perceived by human senses and the breeding background. It is expected that the results of the present study will provide information on the cultivars that have better flavor based on volatile and non-volatile organic components and are most suitable for organic low-input production and as parents in organic breeding.

## Materials and Methods

### Plant Materials, Experimental Layout, and Crop Cultivation

The experiments were carried out with indeterminate cultivars corresponding to the group of salad (average fruit weight, 103 g) and cocktail (average fruit weight, 26 g) cultivars. Morphological, leaf nutrient, fruit yield, and selected fruit quality traits of the tested cultivars have been previously studied (32).

Sixty cultivars (33 salad and 27 cocktail cultivars) were selected, with information from extension services, research stations, breeders, and seed companies in Germany, Austria and Switzerland, and the IPK Genebank 2015 (Supplementary Table 1), a variety reduction selection process was carried out in which 60 cultivars were reduced to a subgroup of 20 cultivars (8 salad and 12 cocktail cultivars). These 20 selected cultivars were the best cultivars in terms of four traits: yield, total soluble solids (TSS), titratable acidity (TA), and the sensory attribute aroma. Reducing the number of cultivars to a few could increase the effectiveness of breeders in further selection of certain desirable traits among genotypes in breeding programs. The 60 cultivars and a subset of 20 cultivars were, respectively, grown in the field under low-input conditions at Reinshof Experimental Station, University of Göettingen, Germany, according to standard organic horticultural practices. In this study, the term “low-input” includes the avoidance of off-farm inputs during cultivation, especially fertilizer application and the use of moderate irrigation. The field had been used for organic farming since 2003, and faba beans and winter wheat were grown as preceding crops in 2015 and 2016, respectively.

The field trial was arranged in a randomized complete block design with eight biological replications (one and two plants per plot in 2015 and 2016, respectively). Further details of cultivation conditions and agronomic treatments are given in Chea et al. (32) and Erika et al. (33).

### Preparation of Samples

Fruits were harvested fully mature between early August and October at two harvest dates [2015: 13 weeks after planting (WAP) and 18 WAP; 2016: 14 WAP and 19 WAP]. The healthy fruit samples were brought to the laboratory and immediately measured for fruit color. A tomato sample consisting of three to ten fruits per biological replication was used for each of the analyses. After washing, it was divided for five subsamples. One subsample was used for the immediate extraction of VOCs. Another subsample was used for sensory evaluation the following day, while the other subsamples were sliced and stored at −20°C before being analyzed for TSS and TA, and further traits.

### Instrumental Analysis

The measurements of fruit color, TSS, and TA were performed according to Kanski et al. (34), whereas VOCs analysis was conducted by headspace solid phase micro-extraction, and subsequent gas chromatography equipped with a flame ionization detector for detection (HS-SPME-GC-FID), following the method of Ulrich and Olbricht (35). Fruits were rinsed with the ionized water, cut into wedges and homogenized in two parts of volume of 20% NaCl solution (w/v), with a hand mixer (Braun, Germany) at medium speed for 2 min. The homogenate was filled in 50-ml centrifuge tubes and centrifuged at 4°C and 3,000 rpm for 30 min (Centrifuge 5804 R, Eppendorf, Hamburg, Germany) to separate clear supernatant. Ten milliliters of the supernatant were mixed carefully with 20-μL internal standard (5 μL 1-octanol dissolved in 10-ml ethanol). Subsequently, an 8-ml aliquot was transferred into a 20-ml headspace vial (Gerstel GmbH, Germany) already containing 4-g NaCl and sealed with a screw cap septum. The VOC extract samples were vortexed for 10 s and stored at −80°C until analysis.

Prior HS-SPME analysis, the frozen VOC samples were incubated for 15 min at 35°C with a shaking operation mode of 300 rpm to allow equilibration of volatiles in the headspace, and then they were exposed to the vial headspace for another 15 min at 35°C under the same continuous shaking after an SPME fiber (100-μm poly-dimethylsiloxane/PDMS; Supelco, Bellefonte, PA) was inserted into the vial. Desorption was performed within 2 min in the splitless mode and 3 min with split at 250°C. An Agilent Technologies 6890 GC equipped with an HP-Wax column (0.25-mm i.d., 30-m length, and 0.5-μm film thickness) and FID were used for separation and detection. Carrier gas was hydrogen using a flow rate of 1.1 ml min^–1^. The temperature program was the following: 45°C (5 min) from 45 to 210°C at 3°C min^–1^ and held 25.5 min at 210°C. All samples were run with two technical replications.

The commercial software ChromStat2.6 (Analyt, Műllheim, Germany) was used for raw data processing. Data inputs for ChromStat 2.6 were raw data from the percentage reports (retention time/peak area data pairs) performed with the software package ChemStation [version Rev.B.02.01.-SR1 (260)] by Agilent. Using ChromStat2.6, the chromatograms were divided in up to 200 time intervals, each of which represented a peak (substance) occurring in at least one chromatogram of the analysis set. The peak detection threshold was set on the 10-fold value of noise. The values are given as raw data (the peak area in counts), which also can be described as relative concentration because of the normalized sample preparation. Afterward, the relative concentrations of VOC values were normalized to the mean total abundance of identified compounds and expressed in norm %, calculated according to the following formula:


$$n\,o\,r\,m\%=\frac{A^{i}}{\sum_{1}^{n}A^{i}}$$

where I = substance i; A^i^ = relative concentration of substance i (dimensionless); and *n* = number of observations (identified VOCs). All the analyses were performed using the Statistica 13.3 package (TIBCO Software Inc., Chicago, United States).

The volatiles were identified by parallel runs of selected samples on an identically equipped GC-MS. The VOCs were identified by comparison of their mass spectra with library entries (NIST 14; the National Institute of Standards and Technology, Wiley, Nbs75k, United States), as well as by comparing RI with authentic references. CAS-numbers of the VOCs (Supplementary Table 2) were retrieved from the web-based chemical search engine^1^ and from the online edition of the “specifications for flavorings” database^2^.

### Sensory Evaluation

Due to the high number of cultivars in 2015, a sensory evaluation, using the breeders’ sensory test (36) was carried out. Three panelists (male, 25–50 years of age) with experience in sensory evaluation of tomatoes, knowledge of the cultivars to be evaluated, and the terminology to be used were trained in preparation for the sensory evaluation. Sensory attributes that contribute to the fruit flavor (fruit firmness, juiciness, skin firmness, sweetness, sourness, aroma), and the acceptability were assessed. The scoring was based on a 9-point scale (0 = minimum intensity and 9 = maximum intensity), with the detailed description of the assessed attributes, following Hagenguth et al. (36) for most of the attributes. Fruit firmness referred to resistance of the pericarp during first chewing, and skin firmness referred to the degree to which the epidermis remained intact during chewing. Juiciness was described as amount of liquid that escapes during initial chewing. The aroma was associated with the retronasal impression of the fruit slices. The samples were labeled with a three-digit code and served to the panelists on transparent plastic trays. To avoid fruit type bias, the different fruit types were evaluated in separate groups.

In 2016, quantitative descriptive analysis (QDA) was used to evaluate the sensory attributes of 20 cultivars. Eleven panelists aged 25–55 years (45% male and 65% female) were trained weekly for 22 months in descriptive evaluation of tomatoes, which involved eight training sessions according to the ISO (International Organization for Standardization) standards 8586 (37). The technical procedure for panel training and sensory evaluation sessions was the same as the protocol of sensory methods described in Daoud et al. (24) with modifications. The sensory attributes used in this panel test were similar to those used in the breeders’ sensory test (36). The panelists were trained in eight sessions during 2-week training prior to the evaluation. The first three sessions introduced general sensory techniques and established the descriptive terms used to characterize flavor (fruit firmness, juiciness, skin firmness, sweetness, sourness, and aroma) and the overall acceptability of tomatoes. In the following sessions, each of the attributes was defined, and different references to represent each attribute were presented to the panelist. During the training session, the panel leader mediated group discussions to reach a consensus. The panelists were asked to rate samples in terms of the intensity of the individual flavor attributes (fruit firmness, juiciness, skin firmness, sweetness, sourness, aroma) and the overall acceptance of each sample on a score sheet with an unstructured line scale (0 = minimum intensity to 100 = maximum intensity). Each analysis was carried out in individual booths in the sensory laboratory at the University of Göettingen under daylight-equivalent lighting conditions that met the specifications of ISO 8589 (38). About 30 min before the evaluation, the samples were removed from storage at 7°C to adjust to room temperature and cut into equal sample sizes or to 1/8 wedges, depending on fruit size. The samples were presented to the panelists in small bowls. Water and plain crackers were provided to cleanse the palates.

### Statistical Analysis

Overall mean abundance of instrumental and sensory data was subjected to one-way ANOVA and Tukey’s test for honestly significant difference (Tukey’s HSD) to determine possible differences in means at *p* < 0.05, *p* < 0.01, and *p* < 0.001. Pearson correlation analysis was performed between and within VOCs significantly associated (*p* < 0.05 and *p* < 0.01) with instrumental and sensory parameters. Principal component analysis (PCA) was conducted to identify the principal components responsible for the most of the variations within the dataset. PCA was performed on the instrumentally determined data and the mean sensory ratings (based on the correlation matrix) over both crop years. All the analyses were performed using the Statistica 13.3 package (TIBCO Software Inc., Chicago, United States).

## Results

**FIGURE 1:**
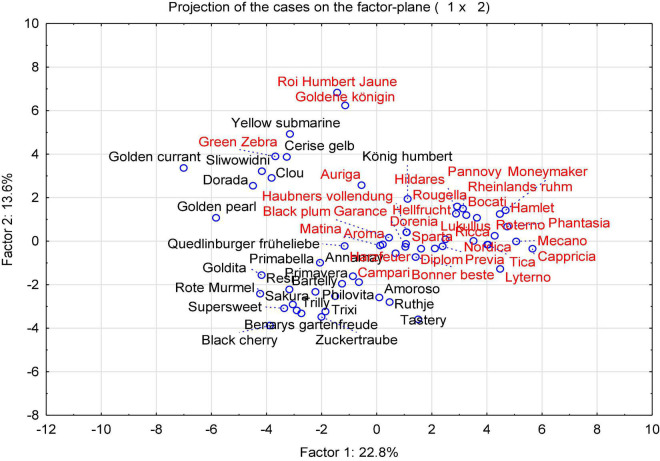
A PCA score plot of the instrumental and sensory attributes of 60 cultivars (33 salad and 27 cocktail cultivars) grown in 2015. Salad cultivars are indicated in red; and cocktail cultivars are shown in black. The full names of cultivar are listed in Supplementary Table 1.

**FIGURE 2:**
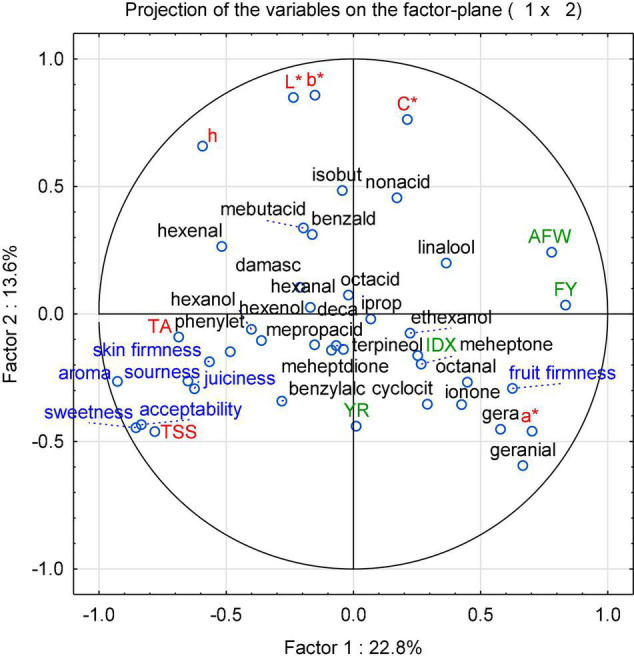
A PCA loading plot of the instrumental parameters (in red and black) and sensory attributes (in blue) as well as other cultivar-related parameters (in green) of 60 cultivars (33 salad and 27 cocktail cultivars) grown in 2015. Instrumental parameters: TSS - total soluble solids; TA - titratable acid; L* – lightness; a* – red (+)/green (−); b*: yellow (+)/blue (−); C* – chroma; h – hue. Sensory attributes: fruit firmness, juiciness, skin firmness, sweetness, sourness, aroma, and acceptability. Other parameters: AFW – average fruit weight, FY – fruit yield, IDX – intensity index, YR – year of release. VOCs are named according to Supplementary Table 2.

With the aim of identifying cultivars best suited for organic low-input production and as parents for organic breeding, the present study assessed tomato fruit flavor quality and its relationship with sensory traits, breeding background and agronomic characteristics, instrumental data, and sensory properties of 60 tomato cultivars. To achieve the objective, 33 salad and 27 cocktail cultivars grown in 2015, and a subset of eight salad and 12 cocktail cultivars grown in 2016 were determined, with a focus on flavor-associated VOCs. The PCA, performed with the mean values of the concentration of 32 identified VOCs (Supplementary Table 2), color parameters, TA, TSS, and sensory data from every year in 2015 and 2016, showed a large diversity (Figure 1). In 2015, all tested samples are distributed across the parameter space, with a more and less distinct clustering, distinguishing mainly cocktail and salad cultivars, with the exception of the salad cultivar “Green Zebra” dislocated in the left quadrant and the cocktail cultivar “König Humbert” in an opposite quadrant. In addition, a separated group in the upper left quadrant of the plot, which included most of the cultivars with yellow and orange fruits as well as with red fruits, could be clearly distinguished (Figure 1). No clear clustering occurs for other subgroups, but a number of cultivars with superior aroma-like “Green Zebra” and “Black Cherry” plotted to the left of the main cluster. The corresponding loading plot (Figure 2) illustrates that fruit firmness, yield (FY), average fruit weight (AFW), intensity index (IDX), and VOCs as geranial and octanal are associated in the cultivars studied. A higher IDX indicates a higher input in the production systems suitable for a cultivar (Supplementary Table 1). Sensory traits regarded as positive (sweetness, sourness, aroma, firmness) clustered together with the related analytical traits TA and TSS, and skin firmness.

### Volatile Organic Compounds of 60 Cultivars

The concentration of VOCs was analyzed in 60 cultivars grown in 2015. Twenty four VOCs were identified in both the salad and cocktail cultivars (Supplementary Tables 3, 4) belonging to five substance classes, such as aldehyde (ALD), ketone (KET), alcohol (ALC), sulfur-derived compound (SDC), and aliphatic acid (ALA). Significant differences were found between cultivars for most VOCs.

**FIGURE 3:**
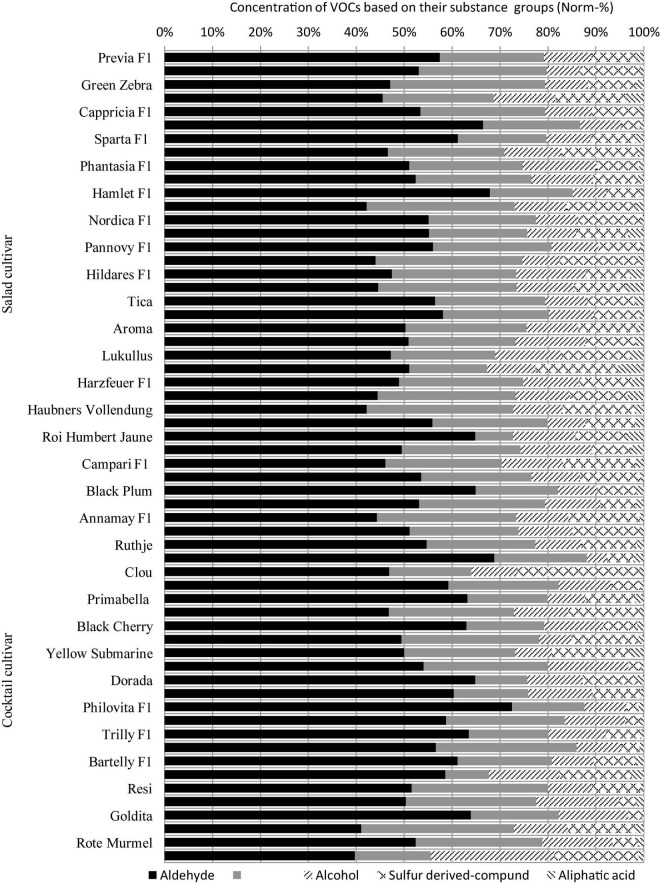
Concentration of VOCs accumulated in the 60 cultivars grown in 2015 compiled to their substance groups. The cultivars are ordered based on average fruit weight from high to low. Cultivar names “Goldene Koenigin”, “Quedlinburger Fruehe Liebe”, and “Koenig Humbert” were written as “Goldene Königin”, “Quedlinburger Frühe Liebe”, and “König Humbert”, respectively. The substance groups of the VOCs: Aldehydes: β-cyclocitral, benzaldehyde, citral, decadienal, (*E*)-2-hexenal, geranial, hexanal and octanal; ketones: 6-methyl-5-hepten-2-one, β-damascenone, β-ionone, (*E*)-geranylacetone, farnesylacetone, and methylheptadione); alcohols: 1-hexanol, 2-ethyl-1-hexanol, α-terpineol, benzyl alcohol, eugenol, linalool, phenylethyl alcohol, and (*Z*)-3-hexen-1-ol; sulfur-derived compound: 2-isobutylthiazole; aliphatic acids: 2-methylpropanoic acid, 3-methylbutanoic acid, octanoic acid, and nonanoic acid; and another substance groups, ester (2-methylbutylacetate, isopropylmyristate, and methylsalicylate) are not included in the figure due to their minor abundance in the tomato fruit samples.

The group of ALD accounted for the largest proportion of the total VOCs in 60 cultivars, followed by KET, ALC, SDC, and ALA. The levels of these chemical groups varied from cultivar to cultivar, with the highest proportions of 72.5, 32.3, 25.8, 25.5, and 5.4% found in “Philovita F1”, “Green Zebra”, “Golden Currant”, “Clou”, and “Goldene Königin”, respectively (Figure 3). Hexanal (ALD) had the highest share of the total measured VOCs in salad and cocktail cultivars, with 27 and 30%, respectively. Methylheptadione (KET) and decadienal (ALD) could only be detected in some cultivars of both groups, while benzyl alcohol (ALC) was present in all salad cultivars and in most cocktail cultivars (Supplementary Tables 3, 4).

### Influence of the Year and Harvest Date on Volatile Organic Compounds

The main effects on the concentration of VOCs in the 20 cultivars grown in 2015 and 2016 are shown in Supplementary Table 5. The effect of cultivar on all identified VOCs, except of α-terpineol, was always significant at different confidence levels. Of the total 31 VOCs identified, the interaction of cultivar (C) × harvest date (H) × year (Y) showed significant effects on 17 VOCs. The VOCs were composed of hexanal (35.2%), 6-methyl-5-heptene-2-one (17.4%), 2-isobutylthiazole (11.1%), (E)-2-hexenal (8.8%), geranylacetone (5.3%), octanal (3.7%), and β-damascenone (3.3%). Other VOCs were detected in relative concentrations of less than 3% of the total quantified VOCs.

The Student’s T-test showed that the relative concentrations of 2-isobutylthiazole, linalool, phenylethyl alcohol, and nonanoic acid were significantly different (*p* < 0.001) between cocktail and salad cultivars, highlighting the differences in tomato aroma between the groups of fruit type. Tables 1, 2 show the variation of concentration for each of the 31 VOCs within the individual salad and cocktail cultivars. There were 15 VOCs in the salad cultivars and 23 VOCs in the cocktail cultivars, each with significant differences at different confident levels. However, these differences were not consistent within the fruit type. For example, hexanal, octanal, and 1-hexanol differed significantly between the cocktail but not between the salad cultivars. Hexanal was predominant in both salad and cocktail cultivars, followed by (*E*)-2-hexenal and octanal. The concentration of hexanal was not significantly different among salad cultivars but varied within the cocktail cultivars, ranging from 25.1% (“Supersweet 100 F_1_”) to 48.7% (“Black Cherry”) of total VOCs. The range of (*E*)-2-hexenal from 7.08 to 13.64% was also high but not statistically different in cocktail cultivars, while significant variation of (*E*)-2-hexenal was found in salad cultivars (3.37–9.94%). Between the fruit type, most of salad cultivars were characterized with higher concentrations of 2-isobutylthiazole, 6-methyl-5-hepten-2-ol, linalool, and 2-isobutylthiazole, while benzyl alcohol and phenylethyl alcohol were found in a considerable level in most of cocktail cultivars (Tables 1, 2).

**TABLE 1:** Concentration of volatile organic compounds (VOCs) (norm-%) of eight salad tomato cultivars grown in 2015 and 2016.

VOCs	Cultivar								Signifi- cance	Tukey’s HSD	Concentration (mean ± SD)	
	Campari F_1_	Auriga	Harzfeuer F_1_	Roterno F_1_	Lyterno F_1_	Bocati F_1_	Cappricia F_1_	Green Zebra			2015	2016
Hexanal	29.60	29.28	31.52	37.43	28.24	36.61	37.01	36.13	ns	20.74	22.4 ± 11.1	44.1 ± 8.60
2-methylbutylacetate	Nd	0.12	Nd	Nd	0.09	Nd	Nd	0.06	***	0.21	0.00 ± 0.00	0.28 ± 1.65
(*E*)-2-hexenal	9.94	9.74	9.03	3.37	6.20	5.79	7.72	9.78	*	5.58	8.86 ± 4.82	6.62 ± 3.16
octanal	4.77	2.93	3.97	3.48	4.86	2.95	4.23	2.30	ns	3.02	5.47 ± 1.75	2.11 ± 0.97
6-methyl-5-heptene-2-one	16.08	13.24	18.75	20.80	17.86	15.90	15.80	24.67	*	9.75	20.7 ± 7.66	15.7 ± 6.13
1-hexanol	1.59	1.65	2.23	1.14	0.82	1.62	0.72	1.31	ns	1.63	1.91 ± 1.38	1.06 ± 0.71
(*Z*)-3-hexen-1-ol	2.85	3.48	3.27	1.49	2.00	1.96	1.65	1.47	ns	2.51	3.59 ± 1.58	1.19 ± 0.95
2-isobutylthiazole	17.32	13.25	11.45	17.88	18.27	17.19	10.82	12.02	***	5.09	13.1 ± 4.04	13.2 ± 5.49
6-methyl-5-hepten-2-ol	0.35	Nd	0.38	0.37	0.55	0.42	0.50	Nd	ns	0.55	0.00 ± 0.00	0.56 ± 0.39
2-ethyl-1-hexanol	0.85	0.72	0.73	0.69	1.05	0.66	0.95	0.68	ns	0.70	0.95 ± 0.58	0.67 ± 0.28
benzaldehyde	0.16	0.28	0.20	0.14	0.10	0.10	0.00	0.14	ns	0.39	0.28 ± 0.33	0.00 ± 0.00
linalool	1.93	2.83	2.48	1.67	3.23	2.98	3.47	2.46	**	2.08	3.51 ± 1.54	1.60 ± 1.04
methylheptadione	0.08	Nd	0.05	0.05	Nd	Nd	Nd	0.02	ns	0.16	0.05 ± 0.15	0.00 ± 0.00
β-cyclocitral	1.03	5.57	1.16	0.97	1.63	0.97	1.47	0.22	***	1.19	2.08 ± 2.08	1.18 ± 0.93
3-mebutanoic acid	0.21	0.48	0.34	0.33	0.19	0.27	0.40	0.55	ns	0.68	0.52 ± 0.55	0.13 ± 0.25
α-terpineol	0.73	0.19	0.30	0.04	0.09	0.11	0.48	0.26	ns	0.99	0.28 ± 0.91	0.27 ± 0.28
geranial	0.94	0.09	1.14	0.98	1.62	0.71	1.17	Nd	*	1.59	1.66 ± 1.21	0.00 ± 0.00
citral	0.75	0.09	0.79	0.72	0.89	0.56	0.37	Nd	***	1.11	0.00 ± 0.00	1.52 ± 1.05
decadienal	Nd	Nd	Nd	Nd	Nd	Nd	Nd	Nd	–	–	0.00 ± 0.00	0.00 ± 0.00
methylsalicylate	0.39	0.51	2.00	0.31	0.07	1.43	0.22	0.66	ns	1.95	0.00 ± 0.00	1.28 ± 1.75
β-damascenone	3.21	5.97	2.35	1.62	3.26	2.25	4.97	5.00	**	3.99	5.34 ± 3.20	1.48 ± 1.48
(*E)*-geranylacetone	4.44	2.77	5.28	4.92	6.07	5.67	5.88	1.11	***	2.75	5.79 ± 2.53	4.49 ± 5.29
2-mepropanoic acid	0.27	0.54	0.24	0.21	0.40	0.15	0.13	0.18	ns	0.56	0.20 ± 0.45	0.27 ± 0.31
benzyl alcohol	Nd	0.09	0.04	Nd	Nd	Nd	Nd	0.05	***	0.17	0.00 ± 0.00	0.16 ± 0.32
phenylethyl alcohol	0.81	0.38	0.20	0.21	0.34	0.19	0.25	0.26	***	0.50	0.38 ± 0.46	0.55 ± 0.60
β-ionone	0.98	4.15	0.95	0.92	1.51	0.82	1.29	Nd	***	1.05	1.80 ± 1.66	0.97 ± 0.74
eugenol	0.19	0.08	0.13	0.05	0.15	0.18	0.03	0.03	***	0.26	0.00 ± 0.00	0.24 ± 0.24
farnesylacetone	Nd	0.64	0.17	0.04	0.08	0.17	0.24	Nd	***	0.37	0.00 ± 0.00	0.34 ± 0.39
isopropylmyristate	Nd	Nd	Nd	Nd	Nd	Nd	Nd	Nd	–	–	Nd	Nd
octanoic acid	0.33	0.50	0.43	0.17	0.42	0.02	0.20	0.14	ns	0.61	0.55 ± 0.49	Nd
nonanoic acid	0.20	0.42	0.41	Nd	Nd	0.35	Nd	0.48	ns	0.67	0.46 ± 0.60	Nd

Each mean represents six biological replicates (over 2 years) and three biological replicates (within 1 year); SD: standard deviation; Nd: not detectable or present at low levels; ns indicates a nonsignificant difference; *, ** and *** indicate significance differences of each factor and interaction at p < 0.05, p < 0.01, and p < 0.001, respectively; HSD (0.05) = critical value for comparisons by Tukey’s honestly significant difference (HSD) tests at p < 0.05.

**TABLE 2:** Concentrations of volatile organic compounds (VOCs) (norm-%) of 12 cocktail tomato cultivars grown in 2015 and 2016.

VOCs	Cultivars												Signifi-cance	Tukey’s HSD	Concentration (mean ± SD)	
	Goldita	Super-sweet 100 F_1_	Resi	Bartelly F_1_	Benarys Garten-freude	Prima-vera	Black Cherry	Sakura	Prima-bella	Tastery F_1_	Annamay F_1_	Amoroso F_1_			2015	2016
Hexanal	30.06	25.11	34.64	40.44	37.16	40.10	48.67	26.26	47.50	42.17	30.31	37.71	***	16.94	29.1 ± 11.2	43.9 ± 9.90
2-methylbutylacetate	Nd	Nd	5.54	Nd	Nd	Nd	Nd	Nd	Nd	Nd	Nd	Nd	***	2.65	0.00 ± 0.00	0.80 ± 3.03
(*E*)-2-hexenal	10.07	12.62	7.25	11.50	7.08	9.23	9.00	13.64	9.59	9.01	7.24	7.92	ns	8.94	12.8 ± 7.07	6.19 ± 2.80
octanal	3.94	4.32	3.26	3.13	3.68	4.19	3.63	3.46	1.90	4.73	3.74	4.53	***	2.34	4.83 ± 1.49	2.44 ± 1.16
6-methyl-5-heptene-2-one	14.61	22.43	22.51	14.26	28.97	13.41	11.48	16.99	10.57	10.80	20.28	17.67	***	6.87	16.7 ± 6.68	17.0 ± 7.52
1-hexanol	2.13	1.35	2.37	1.12	2.25	2.87	2.01	1.38	1.43	1.54	1.71	1.65	***	1.34	2.27 ± 1.08	1.23 ± 0.61
(*Z*)-3-hexen-1-ol	3.53	3.98	2.83	2.04	3.70	4.10	2.45	2.61	2.34	3.16	2.60	2.31	ns	2.94	4.30 ± 2.00	1.48 ± 0.91
2-isobutylthiazole	2.99	3.74	8.74	8.89	3.89	10.52	7.18	14.09	11.41	7.33	16.34	8.06	***	3.39	8.38 ± 4.44	11.1 ± 6.49
6-methyl-5-hepten-2-ol	0.18	0.07	0.12	0.07	Nd	Nd	0.28	0.21	0.29	0.05	0.17	0.09	***	0.27	0.00 ± 0.00	0.30 ± 0.34
2-ethyl-1-hexanol	1.10	0.84	0.54	0.80	0.60	1.22	0.78	0.92	0.55	1.28	0.67	0.96	*	0.69	0.97 ± 0.63	0.72 ± 0.30
benzaldehyde	Nd	0.11	0.07	0.16	0.04	0.04	0.07	0.13	0.02	Nd	0.05	0.21	ns	0.26	0.15 ± 0.24	0.00 ± 0.00
linalool	1.08	4.54	0.65	0.97	0.51	0.25	3.63	2.22	0.53	1.06	1.44	2.30	***	1.41	2.11 ± 1.79	1.17 ± 1.08
methylheptadione	Nd	Nd	Nd	0.10	Nd	Nd	Nd	Nd	Nd	Nd	0.04	Nd	ns	0.12	0.02 ± 0.11	0.00 ± 0.00
β-cyclocitral	0.73	1.84	0.97	1.55	1.17	2.53	0.88	1.56	1.16	2.24	1.19	1.37	***	0.73	1.47 ± 0.76	1.39 ± 0.88
3-mebutanoic acid	Nd	0.14	0.55	0.24	0.16	0.07	0.24	0.08	0.37	Nd	0.12	0.21	**	0.46	0.31 ± 0.42	0.08 ± 0.19
α-terpineol	Nd	0.37	0.14	0.06	0.32	0.84	0.63	0.20	0.06	Nd	0.10	0.32	ns	1.33	0.37 ± 1.23	0.14 ± 0.26
Geranial	0.32	0.81	0.87	0.66	1.03	0.46	0.77	0.80	0.61	1.20	0.57	0.82	ns	1.29	1.48 ± 0.73	0.00 ± 0.00
Citral	0.72	1.94	1.16	1.32	1.51	1.41	0.76	0.81	0.87	0.66	1.64	0.74	ns	1.85	0.00 ± 0.00	1.95 ± 1.17
Decadienal	Nd	Nd	Nd	0.04	0.24	Nd	Nd	Nd	Nd	Nd	Nd	0.05	*	0.21	0.05 ± 0.21	0.00 ± 0.00
Methylsalicylate	0.16	0.08	0.08	0.20	0.54	0.26	0.34	0.14	1.05	2.97	0.13	0.16	***	1.51	0.00 ± 0.00	1.06 ± 1.60
β-damascenone	2.61	4.44	2.80	2.94	0.72	1.50	2.77	6.32	2.32	1.90	4.62	3.83	***	3.70	5.01 ± 2.41	1.37 ± 1.91
(*E*)-geranylacetone	20.17	5.39	3.48	4.43	4.43	4.23	2.63	4.28	4.42	6.42	4.73	5.69	***	3.40	6.19 ± 4.92	4.73 ± 3.57
2-mepropanoic acid	0.22	0.26	Nd	0.36	0.06	0.31	0.40	0.28	0.09	0.47	0.20	0.32	ns	0.63	0.30 ± 0.54	0.23 ± 0.30
Benzyl alcohol	1.25	0.59	0.08	0.47	Nd	Nd	0.24	0.48	Nd	Nd	Nd	Nd	***	0.40	0.16 ± 0.37	0.28 ± 0.50
Phenylethyl alcohol	2.96	3.10	0.26	2.39	0.30	Nd	0.18	1.60	1.14	0.43	0.74	1.29	***	1.00	1.29 ± 1.38	0.96 ± 1.14
β-ionone	0.88	1.35	0.82	1.35	1.12	2.19	0.67	1.23	0.91	2.24	1.08	1.23	***	0.64	1.25 ± 0.72	1.18 ± 0.59
Eugenol	Nd	Nd	0.04	0.11	Nd	0.06	0.06	Nd	0.21	0.30	0.10	0.08	**	0.21	0.00 ± 0.00	0.14 ± 0.20
Farnesylacetone	0.23	0.20	0.19	0.12	0.27	0.07	Nd	0.12	0.14	Nd	0.05	0.12	**	0.34	0.00 ± 0.00	0.25 ± 0.40
Isopropylmyristate	Nd	Nd	Nd	Nd	Nd	Nd	Nd	Nd	Nd	Nd	Nd	Nd	–	–	Nd	Nd
Octanoic acid	0.03	0.36	0.05	0.30	0.26	0.13	0.24	0.20	0.04	0.05	0.16	0.36	*	0.40	0.36 ± 0.33	0.00 ± 0.00
Nonanoic acid	Nd	Nd	Nd	Nd	Nd	Nd	Nd	Nd	0.49	Nd	Nd	Nd	***	0.30	0.08 ± 0.35	0.00 ± 0.00

Each mean represents six biological replicates (over 2 years) and three biological replicates (within 1 year); SD: standard deviation; Nd: not detectable or present at low levels; ns indicates a nonsignificant difference; *, ** and *** indicate significance differences of each factor and interaction at p < 0.05, p < 0.01, and p < 0.001, respectively; HSD (0.05) = critical value for comparisons by Tukey’s honestly significant difference (HSD) tests at p < 0.05.

### Sensory Evaluation

The analysis of sensory characteristics of the tested cultivars focused on the following attributes: texture (fruit firmness, juiciness, and skin firmness), taste (sweetness, sourness), aroma, and overall acceptability. Two types of sensory tests were carried out in order to characterize the fruit quality of the cultivars. In 2015, the breeders’ sensory test was performed on 60 cultivars, whereas, in 2016, a QDA test with a trained panel was used to access the 20 cultivars.

#### Breeders’ Sensory Test of 60 Cultivars

Sensory parameters analyzed on fruits of salad and cocktail cultivars in 2015 varied considerably, with the coefficients of variation (CV) ranging from 15.5 to 26.4 and from 9.6 to 14.8%, respectively (Table 3). Among the salad cultivars, fruit firmness was rated highest for the cultivars “Lyterno F_1_”, “Cappricia F_1_”, “Roterno F_1_”, and “Bocati F_1_”. The highest rating for juiciness was obtained for “Green Zebra”, which were not significantly different from “Harzfeuer F_1_” and “Auriga”, while skin firmness was rated at highest for “Harzfeuer F_1_”, “Auriga”, and “Campari F_1_”. In terms of sweetness, “Campari F_1_”, “Harzfeuer F_1_”, “Green Zebra”, and “Auriga” were evaluated as the sweetest among the salad cultivars, whereas “Green Zebra”, “Auriga”, and “Campari F_1_” had the highest score of sourness. Regarding aroma, “Green Zebra”, “Campari F_1_”, “Auriga”, and “Harzfeuer F_1_” were characterized as the cultivars with the most intensive aroma. In term of overall acceptability among salad cultivars, “Campari F_1_” seems to be the most accepted cultivar by the breeders’ panelists, with the highest value from 5.3 to 6.2 (scale, 0: minimum intensity, - 9: maximum intensity), but these cultivars did not significantly differ from “Green Zebra” and “Auriga”.

Among the cocktail cultivars, almost all of the selected cultivars used for further evaluation in 2016 were the cultivars that performed highest in at least one of the tested sensory attributes. Exemplary, “Sakura F_1_”, “Supersweet 100 F_1_”, “Benarys Gartenfreude”, and “Goldita” were scored at highest for sweetness and acceptability in the breeders’ sensory test in 2015. In spite of these, some cultivars that were not selected for investigation in 2016 also scored high on certain sensory attributes, such as “Trilly F_1_” for juiciness, sweetness, aroma, and overall acceptability. The so-called wild cultivars “Rote Murmel” and “Golden Currant” were highly appreciated in terms of juiciness, taste, and aroma, but not for firmness. “Trilly F_1_” and “Golden Pearl F_1_” were rated as the most acceptable cultivars in 2015 (Table 3).

**TABLE 3:** Breeders’ sensory scores of 33 salad and 27 cocktail tomato cultivars grown in 2015.

Cultivar	Fruit firmness	Juiciness	Skin firmness	Sweetness	Sourness	Aroma	Acceptability
Salad cultivar							
Previa F_1_	4.25	5.67	4.54	4.21	5.33	5.02	4.46
Garance F_1_	4.86	4.48	5.79	4.38	5.75	4.96	4.04
**Green Zebra**	2.86	6.58	4.90	4.94	7.31	6.69	6.09
Diplom F_1_	4.34	6.31	5.46	4.40	4.83	4.29	3.75
**Cappricia F_1_**	6.85	4.98	3.88	2.71	3.79	2.29	2.00
Rougella F_1_	5.98	3.88	5.29	3.31	3.71	3.27	2.33
Sparta F_1_	5.42	6.06	4.96	4.38	5.56	5.12	4.96
**Bocati F_1_**	5.86	5.29	3.56	2.77	4.60	3.10	2.67
Phantasia F_1_	7.35	3.77	4.38	2.60	4.56	2.52	2.00
Mecano F_1_	6.96	4.44	3.79	2.44	3.83	2.17	1.87
Hamlet F_1_	6.65	3.39	4.65	2.33	3.34	2.42	1.92
**Lyterno F_1_**	7.04	5.06	4.63	3.29	5.04	3.48	3.17
Nordica F_1_	6.85	5.08	3.75	3.29	4.17	3.08	2.48
Moneymaker	6.17	4.67	4.94	2.13	3.52	2.02	1.50
Pannovy F_1_	5.06	4.25	4.54	2.50	4.96	2.75	2.46
**Roterno F_1_**	6.46	5.33	3.63	3.52	3.52	3.15	2.73
Hildares F_1_	4.56	5.08	4.96	3.56	4.12	3.54	2.56
Bonner Beste	4.66	6.17	6.45	4.17	5.95	4.83	4.57
Tica	7.02	4.58	4.09	2.79	4.50	2.81	2.54
Ricca	7.23	4.61	4.00	2.60	4.13	2.62	2.23
Aroma	4.92	5.86	5.71	4.54	4.94	4.96	3.94
Rheinlands Ruhm	5.67	5.54	4.79	2.65	3.67	2.56	1.92
Lukullus	5.21	5.19	4.73	3.31	4.42	3.56	2.44
Goldene Königin	3.60	5.08	5.38	3.92	4.54	4.67	3.19
**Harzfeuer F_1_**	4.63	5.69	6.11	5.15	5.10	5.04	4.34
**Auriga**	3.58	5.65	5.67	4.73	6.02	5.94	5.33
Haubners Vollendung	3.37	5.42	5.58	4.29	5.17	4.98	3.50
Dorenia	5.09	5.19	4.90	4.17	5.00	4.54	4.00
Roi Humbert Jaune	3.83	4.56	5.83	3.42	4.46	4.33	3.02
Hellfrucht	4.92	5.85	5.40	3.77	4.56	3.67	3.14
**Campari F_1_**	4.77	5.27	5.56	6.23	5.33	6.25	6.21
Matina	4.83	5.96	6.29	4.91	4.57	4.95	4.43
Black Plum	3.08	3.12	4.83	4.71	3.88	5.21	2.67
Mean	5.3	5.1	4.9	3.7	4.7	4.0	3.3
SD	1.5	1.2	1.1	1.2	1.3	1.5	1.5
CV (%)	15.52	15.87	18.43	20.82	18.8	23.15	26.36
HSD (0.05)	1.68	1.66	1.87	1.58	1.81	1.89	1.77
Significance							
Cultivar (C)	***	***	***	***	***	***	***
Harvest (H)	**	***	ns	*	***	*	***
Interaction (C × H)	***	ns	ns	ns	ns	ns	ns
Cocktail Cultivar							
**Amoroso F_1_**	6.46	5.69	4.08	5.71	5.46	5.38	5.27
**Annamay F_1_**	6.00	5.50	4.81	6.17	5.38	5.93	6.24
Quedlinburger Frühe Liebe	3.56	6.65	5.94	5.19	5.92	5.00	4.00
Ruthje	5.79	6.02	5.44	5.98	5.08	5.40	5.06
König Humbert	3.50	2.58	4.46	4.15	3.42	4.29	1.75
Clou	4.38	6.75	7.04	5.42	5.84	5.88	4.88
**Tastery F_1_**	8.10	5.75	5.13	6.44	4.38	4.38	4.96
**Primabella**	6.27	5.52	5.61	5.73	6.33	5.98	5.42
**Sakura F_1_**	5.88	6.63	5.92	7.48	5.98	7.15	7.44
**Black Cherry**	3.27	6.46	6.92	6.63	6.61	7.73	7.33
Cerise gelb	5.21	4.75	5.52	5.67	5.08	6.06	4.77
Yellow Submarine	3.79	4.48	5.38	5.29	5.04	5.77	3.96
Zuckertraube	4.02	6.75	5.69	6.63	5.73	6.36	6.15
Dorada	4.21	6.44	5.25	6.23	5.54	6.31	6.11
**Primavera**	4.00	6.46	5.63	5.50	5.27	5.21	4.79
Philovita F_1_	6.79	5.54	5.15	6.13	5.10	5.94	6.10
Trixi	5.75	6.04	6.73	6.90	4.73	6.17	5.77
Trilly F_1_	6.65	5.38	5.50	7.58	5.31	6.69	7.17
**Benarys Gartenfreude**	4.63	5.19	7.50	7.13	5.56	6.56	5.44
**Bartelly F_1_**	3.79	5.60	4.90	6.65	5.19	6.65	6.29
Golden Pearl F_1_	4.54	6.63	6.63	7.46	5.12	7.06	7.00
**Resi**	2.69	6.94	6.63	6.36	6.11	7.52	6.15
**Supersweet 100 F_1_**	3.79	6.79	6.06	7.35	6.02	7.19	7.13
**Goldita**	3.88	6.33	6.52	7.10	6.12	7.29	6.96
Sliwowidnij	2.98	6.77	5.96	5.73	5.85	6.10	5.42
Rote Murmel	2.58	7.38	5.02	6.67	5.88	7.04	6.65
Golden Currant	3.33	7.15	4.23	7.06	4.79	6.83	5.46
Mean	4.7	6.0	5.7	6.3	5.4	6.2	5.7
SD	1.6	1.2	1.1	1.0	1.0	1.1	1.4
CV (%)	14.8	10.2	12.4	9.6	11.7	11.0	14.1
HSD (0.05)	1.39	1.23	1.41	1.21	1.28	1.37	1.61
Significance							
Cultivar (C)	***	***	***	***	***	***	***
Harvest (H)	ns	***	**	***	***	**	**
Interaction (C × H)	*	***	ns	**	ns	**	ns

The breeders’ sensory scoring was based on a 9-point scale where 0 = not detectable and 9 = maximum intensity; mean values are given for each of the 33 salad cultivars and the 27 cocktail cultivars as mean from samples grown in 2015; SD = standard deviation; CV = coefficient of variation; ns indicates a nonsignificant difference; *, ** and *** indicate significance differences of each factor and interaction at p < 0.05, p < 0.01, and p < 0.001, respectively; HSD (0.05) = critical value for comparisons by Tukey’s honestly significant difference (HSD) test at p < 0.05. The cultivars are arranged in descending order according to their average single fruit weight. The cultivars shown in bold were selected in 2015 for further evaluation in 2016.

#### Panel Sensory Test of Selected 20 Cultivars

The CV of sensory attributes analyzed in the fruit of salad and cocktail cultivars in 2016 ranged from 5.7 to 17.3 and 5.4 to 12.0%, respectively (Table 4). In terms of the highest rated cultivars, the results of the sensory panel in 2016 were relatively similar to those of the breeders’ sensory test in 2015, e.g., “Supersweet 100 F1”, “Benarys Gartenfreude”, “Sakura F_1_”, and “Bartelly F_1_” were ranked as those with the highest acceptability by both sensory panels. In addition, “Supersweet 100 F_1_”, “Sakura F_1_”, “Bartelly F_1_”, “Goldita”, and “Black Cherry”, which received the highest score of aroma in the sensory panel test, were also rated as the most aromatic cultivars by the breeders’ sensory team. In contrast, “Primavera” had the lowest scores for aroma and acceptability, indicating the least appreciated cultivar among the studied cocktail cultivars.

**TABLE 4:** Panel sensory scores of eight salad and 12 cocktail tomato cultivars grown in 2016.

Cultivar	Fruit firmness	Juiciness	Skin firmness	Sweetness	Sourness	Aroma	Acceptability
*Salad cultivar*							
Green Zebra	25.01	72.02	43.02	30.80	61.60	53.24	46.01
Cappricia F_1_	62.61	57.09	36.10	17.76	32.63	26.08	22.09
Bocati F_1_	55.46	60.71	38.70	20.41	31.01	28.41	22.38
Lyterno F_1_	63.91	58.93	40.33	20.68	32.95	32.00	26.95
Roterno F_1_	57.70	60.14	35.51	21.20	26.54	28.43	25.40
Harzfeuer F_1_	33.58	57.96	49.10	32.30	37.29	39.99	27.75
Auriga	29.93	63.02	56.33	32.42	56.64	45.46	33.31
Campari F_1_	43.86	59.83	45.18	36.09	44.42	46.17	43.32
Mean	46.5	61.2	43.0	26.5	40.4	37.5	30.9
SD	15.3	5.7	8.1	7.9	12.6	10.3	10.1
CV (%)	9.4	5.75	11.55	16.91	7.17	10.1	17.31
HSD (0.05)	7.89	6.35	8.97	8.08	5.23	6.83	9.66
Significance							
Cultivar (C)	***	***	***	***	***	***	***
Harvest (H)	*ns*	*ns*	*ns*	*ns*	*ns*	*ns*	*ns*
Interaction (C × H)	*ns*	*ns*	*ns*	*ns*	*	*	*ns*
*Cocktail cultivar*							
Amoroso F_1_	54.39	60.08	39.97	45.59	41.16	50.73	51.98
Annamay F_1_	55.68	59.78	45.23	40.75	46.35	51.50	49.24
Tastery F_1_	78.02	59.98	45.12	51.08	26.92	40.49	45.08
Primabella	49.28	62.35	42.86	38.54	49.63	51.29	45.69
SakuraF_1_	43.90	61.39	49.15	48.09	47.96	57.08	56.56
Black Cherry	33.09	63.61	53.03	42.00	55.06	54.79	51.10
Primavera	26.22	67.67	50.35	41.81	37.36	43.87	35.36
Benarys Gartenfreude	43.21	57.81	56.94	46.13	46.20	51.82	42.00
Bartelly F_1_	32.30	57.15	45.70	48.85	41.21	56.85	52.88
Resi	29.80	66.22	53.53	42.87	49.62	54.32	45.59
Supersweet 100 F_1_	29.93	58.89	54.03	53.94	46.18	59.11	57.47
Goldita	28.41	59.96	56.88	47.53	48.54	55.58	49.15
Mean	42.0	61.2	49.4	45.6	44.7	52.3	48.5
SD	15.5	4.7	6.5	7.2	8.4	6.3	8.0
CV (%)	10.68	5.42	7.7	12.05	10.46	6.11	9.96
HSD (0.05)	8.47	6.27	7.18	10.38	8.83	6.04	9.13
Significance							
Cultivar (C)	***	***	***	***	***	***	***
Harvest (H)	***	**	*	***	***	**	*ns*
Interaction (C × H)	*ns*	**	*ns*	*ns*	*ns*	**	***

The panel sensory was rated based on a 0 to 100 sensory perceptible scale (0 = minimum intensity and 100 = maximum intensity); mean values are given for each of the eight salad and the 12 cocktail cultivars as mean from samples grown in 2016; SD = standard deviation; CV = coefficient of variation; ns indicates a nonsignificant difference; *, ** and *** indicate significance differences of each factor and interaction at p < 0.05, p < 0.01, and p < 0.001, respectively; HSD (0.05) = critical value for comparisons by Tukey’s honestly significant difference (HSD) test at p < 0.05. The cultivars are arranged in descending order according to their average single fruit weight.

### Correlations Between Traits

The correlations between all traits based on 60 cultivars are given in Supplementary Table 6. The year of release did not have significant influence on quality traits with few exceptions: the content of nonanoic acid decreased; TSS, fruit firmness, and acceptability slightly increased. A significant negative correlation of quality traits with yield was observed. The compounds octanal, linalool, geranial, β-ionone showed positive correlation with yield; whereas (*E*)-2-hexenal, 1-hexanol, (*Z)*-3-hexen-1-ol, and phenylethyl alcohol were negatively correlated with yield formation. Yield and intensity index correlated positively with fruit firmness and negatively correlated with skin firmness.

For the 20 cultivars, significant correlations between the VOCs and instrumental and sensory traits are shown in Table 5. Of the 31 VOCs, 12 compounds were correlated with at least one sensory attribute. Phenylethyl alcohol and (*E*)-2-hexenal, both strongly correlated with TSS, were also positively correlated with sweetness, aroma, and acceptability (*r* > 0.55), whereas 2-isobutylthiazole and 6-methyl-5-hepten-2-ol were negatively correlated with these sensory traits. Both geranial and 6-methyl-5-hepten-2-ol showed a negative correlation with TA and sourness. Geranial was positively correlated (*r* > 0.60) with fruit firmness and negatively with juiciness. The strongest positive correlation was determined between (*Z*)-3-hexen-1-ol and skin firmness (*r* = 0.80). Among instrumental traits, the strongest negative correlation was found between 2-isobutylthiazole and TSS content (*r* = −0.68). A few correlations were significant between VOCs and color components. For example, citral correlated negatively with fruit color Parameters L* and b*, and geranial correlated positively with h value. In addition, Supplementary Table 7 shows that some correlations between VOCs were also observed, such as β-ionone, which had the strongest positive correlation with β-cyclocitral (*r* = 0.98), followed by phenylethyl alcohol, which correlated positively with benzyl alcohol (*r* = 0.83).

**TABLE 5:** Correlation coefficients between volatile organic compounds (VOCs) and instrumental parameters and between VOCs and sensory attributes in the 20 cultivars grown in 2015 and 2016.

VOCs	Instrumental parameters^#^							Sensory attributes						
	TSS	TA	L*	a*	b*	C*	h	Fruit firmness	Juiciness	Skin firmness	Sweetness	Sourness	Aroma	Acceptability
(*E*)-2-hexenal	0.59**	0.54*	–0.10	–0.33	–0.03	–0.18	0.23	−0.49*	0.12	0.48*	0.64**	0.50*	0.67**	0.69**
octanal	0.00	−0.45*	–0.35	0.23	–0.32	–0.33	–0.30	0.34	−0.50*	–0.04	0.11	−0.51*	–0.22	–0.07
1-hexanol	0.31	0.22	–0.19	–0.12	–0.20	–0.29	0.02	−0.49*	0.38	0.65**	0.39	0.26	0.33	0.12
(*Z*)-3-hexen1-ol	0.54*	0.27	–0.15	0.04	–0.08	–0.17	–0.09	–0.39	0.00	0.80**	0.58**	0.20	0.37	0.26
2-isobutylthiazole	−0.68**	–0.41	0.16	0.31	0.22	0.37	–0.17	0.34	–0.03	−0.61**	−0.69**	–0.31	−0.55*	−0.49*
6-methyl-5-hepten-2-ol	−0.52*	−0.56*	–0.11	0.51*	–0.12	0.07	–0.42	0.44	−0.45*	0.52*	−0.69**	−0.48*	−0.64**	−0.59**
3-methylbutanoic acid	−0.45*	0.13	0.44	–0.09	0.39	0.47*	0.21	–0.25	0.36	–0.18	−0.50*	0.28	–0.15	–0.29
geranial	–0.08	−0.60**	−0.53*	0.58**	0.51*	–0.34	−0.66**	0.63**	−0.67**	–0.30	–0.19	−0.68**	−0.47*	–0.35
citral	0.64**	0.08	−0.67**	0.34	−0.60**	−0.53*	−0.54*	–0.11	–0.25	0.35	0.55*	–0.07	0.37	0.35
benzyl alcohol	0.51*	0.40	0.10	–0.24	0.08	–0.05	0.24	–0.43	–0.06	0.49*	0.49*	0.29	0.52*	0.49*
phenylethyl alcohol	0.63**	0.41	–0.10	–0.04	–0.03	–0.09	0.00	–0.27	–0.25	0.31	0.6**	0.20	0.56**	0.63**
eugenol	–0.24	–0.41	–0.10	0.28	–0.07	0.01	–0.24	0.55*	–0.21	–0.39	–0.15	–0.40	–0.36	–0.22
farnesylacetone	0.01	0.21	0.51*	0.18	0.55*	0.53*	0.02	–0.27	–0.13	0.42	–0.07	0.25	–0.01	–0.16
nonanoic acid	–0.35	0.25	0.5*	–0.16	0.54*	0.57**	0.33	–0.22	0.33	–0.06	–0.37	0.33	–0.11	–0.24

Data used for the Pearson correlation are derived from each of the 20 cultivars as mean from both years. ^#^The instrumental parameters used for Pearson correlation analysis were taken from Chea et al. (33). Significant correlation is indicated by asterisks: *p < 0.05 and **p < 0.01. VOCs – volatile organic compounds; TSS – total soluble solid; TA – titratable acids; color parameters: L* – lightness; a* – red (+)/green (–); b* – yellow (+)/blue (–); C* – chroma; h – hue. Sensory attributes: fruit firmness, juiciness, skin firmness, sweetness, sourness, aroma and acceptability. Only the VOCs, which have significant correlations with at least one of the other parameters, are shown.

### Principal Component Analysis of the Instrumental and Sensory Parameters in 20 Cultivars

**FIGURE 4:**
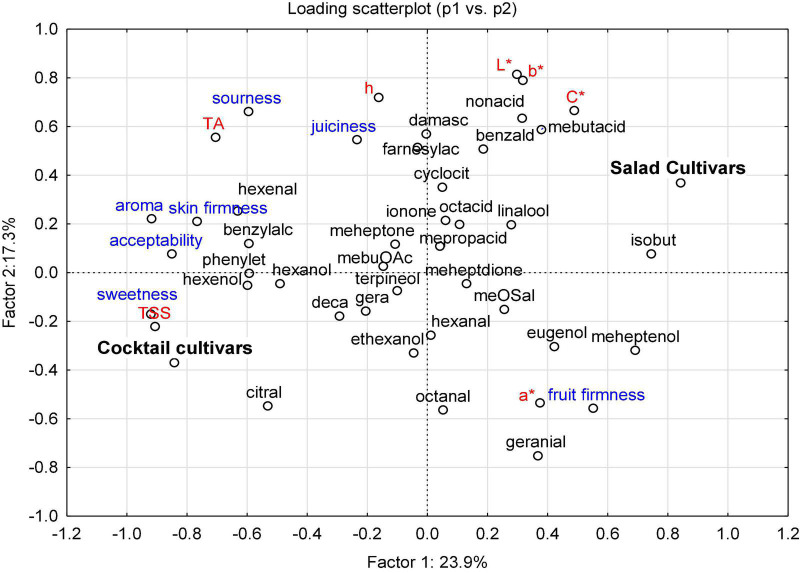
PCA bi-plot of the instrumental parameters (in red and black) and sensory attributes (in blue) of the 20 cultivars (eight salad and 12 cocktail cultivars) grown in 2015 and 2016. Instrumental parameters: TSS – total soluble solids; TA – titratable acid; L* – lightness; a* – red (+)/green (–); b* – yellow (+)/blue (–); C* – chroma; h – hue. Sensory attributes: fruit firmness, juiciness, skin firmness, sweetness, sourness, aroma, and acceptability. VOCs are named according to Supplementary Table 2.

Classification between fruit types, the relative importance of each variable (instrumental and sensory traits), and the relationships between these variables and fruit type are shown in Figure 4. The cultivars were classified into four PCs with respect to their instrumental and sensory characteristics: (i) PC1 distinguished the characteristics of cocktail cultivars (i.e., “Goldita” and “Supersweet 100 F_1_”) from salad cultivars (i.e., “Bocati F_1_”, “Cappricia F_1_”, “Lyterno F_1_”, “Roterno F_1_”), (ii) PC2-contained data of “Auriga” and “Green Zebra”, and separated these salad cultivars from others in the same PC, (iii) in PC3, “Resi” was distinguished from other cultivars, and PC4 contained only “Tastery F_1_” differed from the other CVs (Supplementary Table 8). PC1 was assigned a higher score for TSS, sweetness, aroma, and acceptability, as well for variation in 2-isobuthylthiazole, 6-methyl-5-hepten-2-ol, (*E*)-2-hexenal, (*Z*)-3-hexen-1-ol, phenylethyl alcohol, and benzyl alcohol. High loading of TA; color parameters (L*, b*, C*, and h); sensory attributes (sourness and juiciness); and a number of VOCs, including nonanoic acid, 3-mebutanoic acid, β-damascenone, farnesylacetone, and benzaldehyde, contributed to the separation of PC2. While PC1 and PC2 were generally associated with higher scores of sensory attributes, PC3 and PC4 were distinguished from the other PCs mainly by higher loading values of VOCs (Supplementary Table 9). Overall, the classification of the two fruit types was mainly influenced by the highest loading score of “sweet-taste factor” (i.e., TSS/sweetness), the intensity of aroma, and the flavor-related VOCs mentioned above.

## Discussion

Fruit flavor is a complex trait influenced by interaction of both fruit biochemical characteristic and consumer sensory perception (35, 39). In terms of fruit quality, high variability was observed between tomato cultivars, with salad cultivars having 10–70% lower values for minerals, dry matter, TSS, and total phenolics than cocktail cultivars (31).

### Volatile Organic Compounds Profile of the Tomato Cultivars

A total of 31 VOCs, with aldehydes accounting the largest proportion of the total VOCs concentration, were detected in the samples. This result is in agreement with Wang et al. (40), who reported that ALD accounted for the highest proportion of the total VOCs concentration in tomato fruits, followed by ALC and KET, with all three compound classes compromising more than 95% of total VOCs concentration. Selli et al. (41) reported that 77.2% of the total VOCs in tomatoes were ALD. Of more than 400 VOCs identified in tomato fruits (42), most studies agreed that about 16 VOCs were likely to contribute significantly to tomato aroma (14, 18, 43). Of these, nine VOCs were identified in this study, which are believed to contribute to tomato aroma, such as ALD (hexanal and trans-2-hexenal), ALC (cis-3-hexenol and 2-phenylethyl alcohol), KET (6-methyl-5-hepten-2-one, β-damascenone, and β ionone), SDC (2-isobutylthiazole), and EST (methylsalicylate).

In both years, hexanal derived from C6 fatty acid was the most abundant VOC found, with a mean value of 35.05%. Hexanal is often associated with the perception of “green” and “grassy” in tomato fruits (41), as well as with the evaluation of sweetness of ripe tomatoes (44). The open chain apocarotenoid cleavage product, 6-methyl-5-heptene-2-one, derived from lycopene (14), was the second most abundant VOC (17.77%), followed by the branched chain amino acid-derived VOC 2-isobutylthiazole, with a relative concentration of 11.37%. The latter is one of the most important and interesting aroma components of tomato, as its concentration remains stable during fruit ripening (20) and is not affected by crushing and exposure to oxygen (45). Two esters, namely, farnecylacetone and isopropilmyristate have been identified in the VOCs of the tomato fruits. The latter was detected in trace amounts, and its concentration was not influenced by the interaction of cultivar and environmental factors (Supplementary Table 5). This is in agreement with Goulet (46), who reported that red-fruited tomato cultivars have relatively low levels of acetate esters compared with green-fruited cultivars. In the present study, farnecylacetone was shown to correlate positively with color (L*, b*, and C* values), but not with sensory perceptions (Table 5). However, their overall impact on sensory properties is negligible, and they are not relevant to tomato aroma (16). The fruity esters that are important for flavor in most fruits, e.g., in strawberry (47) do not seem to have the same role in tomato fruits, which may explain the lack of contribution and relevance of esters for sensory evaluation of flavor (16, 46).

### Cultivar and Harvest Season Effects on Instrumental Traits

Breeders have made considerable efforts to improve the quality characteristics of tomato. The suitability of cultivars for a particular location may not be similar to another location (48). Usually, inconsistent quality performance in different growing environments is the result of genotype by environment (G × E) interaction (GEI) (49). Cultivar-by-harvest and year (C × H × Y) interactions were significant for 17 VOCs and not significant for 14 VOCs (Supplementary Table 5), illustrating the stability performance of the cultivars at different harvest dates, years, and their interactions. The information would facilitate the selection of stable/unstable genotypes that perform well in different environments (39). Generally, breeders use GEI to select genotypes with high and stable performance in different environments, and the genotypes whose GEI is insignificant are considered stable (50).

The wide cultivar variation in fruit VOCs concentration in salad and cocktail cultivars (Tables 1, 2) indicates that some cultivars could be used as parents to develop well-adapted cultivars with improved flavor in different environments, such as harvest date (H) and year (Y) in organic low-input management. The effect of cultivar alone was much more important, and it was significant for almost all the compounds, excluding α-terpineol. Paolo et al. (8) and Rambla et al. (16) reported that, although oxygenated terpenoides are among the most abundant volatiles in vegetative tissues of tomato plants and, particularly, in trichomes, only a few of them, including α-terpineol, are present in the ripe fruit, and their influence on fruit flavor is negligible.

The significant C × H × Y interaction effects on VOCs underline the need in breeding for these traits, as plant breeders have to develop cultivars that perform consistently well under different environmental conditions and seasons. Bauchet and Causse (51) reported that VOCs exhibiting a variable pattern of heritability indicate a high sensitivity of these compounds to environmental conditions. As an example, even when fruits of the same genotypes are grown with identical field management, consumers usually complain that off-season tomato fruits are not as good as in-seasonal ones in terms of overall flavor and eating quality (22). This can be a problem for plant breeders as it is too labor-intensive to develop cultivars for each specific site (48) or each specific growing season.

In our previous study, no C × Y interaction was found for TSS and TA within both salad and cocktail cultivar groups (32). This result is consistent with other studies (10, 52) and thus indicates a high stability of these taste-related traits to environmental variation. This is also in line with Gautier et al. (53), who found that the influence of temperature and irradiance on the level of secondary metabolites was more pronounced than on the primary metabolites (e.g., TSS). Based on heritability and G × E interaction, TA was the least environmentally sensitive trait and was much more likely to be retained when the cultivar was grown in a different location (50). Basically, the extent of a G × E interaction is influenced by the genetic structure of the genotype (54).

The fruit color analyzed instrumentally was less variable as the fruits of most cultivars were red (a* > 10), with exception of the salad cultivar “Green Zebra” (green – yellow) and the cocktail cultivar “Black Cherry”, which was characterized by a red – brown fruit skin color (32). The ALD geranial correlated positively with a* and b* levels, while the ALD citral and the EST farnecyl acetone showed a positive relationship with b* (Table 5), so there seems to be a relationship between these VOCs and carotenoids. Genotype is an important determinant of the extent of variability in carotenoid content of ripe tomato fruits (12). The red color value (a*) of our cultivars was lower than that of the cocktail cultivars studied in Sonntag et al. (55), while the color values of the red-fruited salad cultivars (“Bocati F_1_”, “Cappricia F_1_”, “Roterno F_1_”) were comparable to the results of Sonntag et al. (55). The red color of the tomato fruits is due to the synthesis of lycopene and degradation of chlorophyll (56), while an orange genotype accumulates high levels of β-carotene in addition to a low-lycopene content (57). Increasing a* value, lycopene and ß-carotene were observed during ripening of red-fruited cultivars (55). Based on heritability and genotype by environment interaction, lycopene, which accounts for more than 85% of total carotenoids in many red-fruited cultivars (58), was the most environmentally sensitive trait (50). The difference in the levels of a* and b* in the cultivar (32) could be due to the accumulation of derivatives of carotenoid metabolism, such as C8-ketone 6-methyl-5-hepten-2-one, C10-aldehyde geranial, β-ionone, and β-damascenone, which were present in higher concentration in the studied cultivars (Tables 1, 2). With the exception of geranial, these carotenoid derivatives did not significantly influence the flavor perception of the fruit, despite their high content. The higher amount of 6-methyl-5-hepten-2-one might be mainly due to a higher lycopene content in the pericarp (40); however, there was no significant correlation between the amount of the carotenoid derivate and the sensory attributes in our study.

Among the salad cultivars, “Campari F_1_” had the highest TSS, and “Green Zebra” yielded the highest TA. In the cocktail cultivars, “Benarys Gartenfreude” gave the highest value of taste components, followed by “Supersweet 100 F_1_” [see data in Chea et al. (32)]. The TSS, commonly used as an indirect measure of sugar content and sweetness, is considered to be the trait with the highest influence on cherry tomato purchase preference (30). The fact that fruit size and yield per plant are lower in cocktail cultivars than in salad cultivars (32) is, probably, a possible explanation for the higher concentration of TSS observed in this fruit type group. In contrast, TA levels do not seem to be related to fruit type. From a commercial perspective, the content of organic acids in fruits is one of the most important characteristics influencing the sensorial qualities of the product (31).

### Sensory Characteristics of the Cultivars

Improving the sensory quality of fresh market tomato is of great interest to breeders, but it is a very complex goal (6). From the breeder’s perspective, analysis of aroma compounds in fruit crops demands expensive equipment and training (i.e. sensory analysis). Although breeders can use sensory analysis, it is often difficult to perform and requires access to a panel and considerable expertise (18). In the present study, breeders’ sensory test was used as a tool to evaluate organoleptic quality in 2015, as a breeders’ sensory panel is better suited to evaluate a large number of small samples with a small team. Despite their obvious advantages regarding number of cultivars and, more importantly, the time required for analysis, field evaluations are generally subjective and prone to error because they are usually based on the sensory preferences of one or few individuals (39). However, Hagenguth et al. (36) demonstrated a highly significant correlation of sweetness and TSS and sourness and TA, indicating the efficiency of the breeders’ sensory test. A trained panel may not able to assess a large number of samples in a short time, which is typical for early breeding generations, for example, that also require replications (36).

Sensory quality of tomato fruit thus includes taste, aroma, and texture (referred to as flavor), as well as color (24). It seems that a high rating of sensory aroma and measured a* value were important factors that enhanced the acceptability of “Supersweet 100 F_1_” and “Sakura F_1_”, regardless of their superiority in sweetness as the most important criterion. Red-colored tomatoes are more familiar and attractive to consumers than other colored fruits (59, 60). Regarding the aroma, both sensory tests confirmed that “Supersweet 100 F_1_”, “Black Cherry”, “Sakura F_1_”, and “Goldita” had the most intensive aroma. Zörb et al. (58) found that, besides aroma, other sensory attributes, such as sweetness, juiciness, and a fruit-like appearance, are more important for cocktail tomatoes, which are usually eaten raw. The highest scores for aroma and juiciness were obtained for “Black Cherry”, which performed best in the sensory tests in 2015 and 2016 (Tables 3, 4). “Black Cherry” is known as a traditional or “heirloom” type and is usually appreciated for its distinct aroma, especially compared to modern cultivars (21, 61). The high consumer acceptance of “heirloom” tomatoes is due to their excellent fruit quality in terms of TSS, TA, TSS/TA ratio, and sensory properties in terms of sweetness, sourness, and tomato-like taste (62). On the other hand, the increasing demand for “heirloom” cultivars is often associated with a better flavor, offering a “taste of the past” that modern cultivars lack (63). “Black Cherry” not only performed best in terms of aroma but also featured a unique red-deep brown fruit color. Barry and Pandey (64) suggested that the red-brown color of a tomato fruit may be the result of the retention of chlorophyll and the simultaneous accumulation of lycopene during fruit ripening. It was also interesting that the two sensory tests showed a consistent result for all attributes studied. Across all tested cultivars, “Supersweet 100 F_1_”, “Sakura F_1_”, and “Black Cherry” were characterized by very satisfactory overall performance and were superior to other cultivars in terms of sensory quality.

### Correlation Between Traits

Correlations between all traits were calculated to identify relationships between these parameters (Figure 2 and Table 5). No general trend that indicated a superiority of older cultivars was observed, although senior consumers often believe that tomatoes “tasted better in the past.” The cultivars used in this study derived from largely diverse backgrounds, including breeding for intensive indoor production to low-input organic management. Particularly, growers using low-input systems frequently concentrate on high organoleptic quality. This may be the reason why important sensory and other traits do not depend on the age of a cultivar.

Although hexanal had the highest concentration in the cultivars, it did not show a significant correlation with other traits, confirming that the compounds with high abundance do not always necessarily characterize the fruit aroma (65). However, Cebolla-Cornejo et al. (52) pointed out that hexanal is one of most important VOC contributing to tomato aroma. The Pearson correlation coefficient was used to infer which VOCs contribute to each sensory attribute (Table 5). Although several compounds have been shown to contribute to other instrumental parameters and sensory traits, our results highlight that few VOCs, including (*E*)-2-hexenal, phenylethyl alcohol, and benzyl alcohol, are important flavor components. Wang and Seymour (21) reported a lower concentration of (*E*)-2-hexenal in tomato, which they consider as a reason of poor aroma in modern tomato cultivars and which is confirmed by the present study. Colantonio et al. (39) found that fatty-acid-derived VOCs [e.g., (*E*)-2-hexenal and (*Z*)-3-hexen-1-ol] and 2-phenylethanol had positive contribution to sweetness perception of the tomato, which was also observed in our study. Also, an apparent negative correlation was found between geranial and TA, and geranial and sourness, but not with overall acceptability. Geranial was reported as one of the volatile compounds that contribute to perceived sweetness independent of sugar concentration and, therefore, strongly associated with tomato flavor intensity (19). This suggests that consumer acceptance of tomatoes could be improved by selecting for optimal concentrations of these VOCs in the fruit. Despite their high relevance as a flavor compound, acids were reported to be less important than sugars in improving overall liking in tomato (39) and strawberry (47).

The compound 2-isobutylthiazole correlated negatively with sweetness (Table 5), which was also shown by Vogel et al. (17), who found that increasing levels of 2-isobutylthiazole correlated with TSS content and lower perception of sweetness. The concentration of 2-isobutylthiazole was lower in the cocktail cultivars than in the salad cultivars. Our result is in line with that of Ursem et al. (66), who found a lower relative content of 2-isobutylthiazole in cherry tomatoes compared to cultivars with higher fruit weight (i.e., beef and round tomatoes). In tomato homogenate, 2-isobutylthiazole was reported as bitter/pungent (67, 68). Although 6-methyl-5-hepten-2-one had the highest concentration after hexanal (Tables 1, 2), a Pearson correlation analysis showed no statistical correlation with sensory attributes. The lack of this correlation in the present study is interesting, as this VOC has typically been associated with fruit-like flavor and overall acceptability (18, 69). Also, negative correlations were found between 6-me-5-hepten-2-ol and all sensory attributes, except fruit firmness. This result was in contradiction with the results from another study (70) that identified 6-me-5-hepten-2-ol as corresponding aromatic alcohol of 6-me-5-hepten-2-one, which was detected only in overripe tomatoes. It was suggested that these two compounds were carotenoid-related VOCs, whose accumulation in the tomato fruit was indirectly affected by ethylene (71). Although 6-methyl-5-hepten-2-one had the highest concentration after hexanal (Tables 1 and 2), a Pearson correlation analysis showed no statistical correlation with sensory attributes. The lack of this correlation in the present study is interesting, as this VOC has typically been associated with fruit-like flavor and overall acceptability (18, 69). Also, negative correlations were found between 6-me-5-hepten-2-ol and all sensory attributes, except fruit firmness. This result was in contradiction with the results from another study (70) that identified 6-me-5-hepten-2-ol as corresponding aromatic alcohol of 6-me-5-hepten-2-one, which was detected only in overripe tomatoes. It was suggested that these two compounds were carotenoid-related VOCs, whose accumulation in the tomato fruit was indirectly affected by ethylene (71). Thus, lower levels of 2-isobutylthiazole and 6- me-5-hepten-2-ol could lead to higher sensory scores, especially for sweetness, sourness, and the aroma, as well as to higher acceptance of the cultivars as found in both sensory tests. Phenylethyl alcohol, reported in a previous study as the most important metabolite to distinguish between cherry and non-cherry cultivars (66), was positively correlated with TSS content, sweetness, aroma, and overall acceptance. Benzyl alcohol was described as an important flavor component that has been shown to contribute to overall liking and flavor intensity of the tomato (39). The lack of high correlation coefficients between VOCs and sensory attributes can be partly due to complex synergistic and antagonistic interaction dynamics of chemical constituents with human receptors, which can alter the level to which individual chemicals are detectable during sensory assessment, especially in complex food crops, such as tomatoes (72).

Pearson correlation analysis of the VOC concentrations across all cultivars revealed that some correlations between metabolites were supported by the biochemical pathway from which they originated. For example, there were strong associations between the apocarotenoid-derived (β-cyclocitral and β-ionone) and the phenylalanine-derived VOCs (phenylethyl alcohol and benzyl alcohol). Apocarotenoid-derived VOCs are synthesized by the oxidative cleavage of carbon–carbon double bonds in carotenoids (73), and their contents increase substantially during the conversion of chloroplasts into chromoplasts (74). They were, in addition, described as fruity and/or floral and are commonly not abundant in tomato fruits but have very low-odor thresholds that allow humans to perceive their odor characteristics (75). Positive correlation between phenylethyl alcohol and benzyl alcohol, which were assigned to phenylpropanoids metabolic pathways originated from phenylalanine (76), was confirmed in our study. Phenylalanine-derived VOCs were expected to contribute to the increase of the perception of sweet taste and overall liking (65). Colantonio (39) highlighted the important role of VOCs as part of the fruit flavor profile in the perception of sweetness, and, therefore, not only sugar acid parameters but also selected VOCs should be included in breeding programs.

Although Vogel et al. (17) (showed a positive relationship between β-cyclocitral and β-ionone and taste-related traits and acceptability, no correlation was found between the accumulation of these VOCs and cultivar acceptability, neither in the breeder’s nor in the sensory panel tests. These contradictory results may be a result of the particular sets of tomato genotypes that were analyzed.

The apparent positive correlation of yield and intensity index with fruit firmness (*r* = 0.53 and *r* = 0.49) and negative correlation with skin firmness may be biased by the assessment. This implied that fruits of a high yielding-cultivar were firmer than those of lower-yielding cultivars, but not for the skin. The results could be biased by the assessment, as a similar correlation trend was not confirmed for the skin firmness. While the firmness of the fruit (i.e., the pericarp) can be well distinguished between the tester’s teeth in the sensory assessment, the skin (i.e., the epidermis) breaks more easily when supported by a firm pericarp and may, therefore, appear to be less firm.

### Principal Component Analysis Between Instrumental and Sensory Data

The location of the sensory attributes in the loadings plot (Figure 2) shows very close correlations between the attributes sweetness, aroma, and acceptability, while the attribute fruit firmness was located in the opposite quadrant of the plot. The results of PCA are consistent with the Pearson correlation analysis (Supplementary Table 6). The acceptability of the cultivars is positively correlated with the sensory attributes sweetness (*r* = 0.94) and aroma (*r* = 0.93). Fruit firmness is negatively correlated (*r* = 0.34), while the other sensory attributes, such as sensory sourness, juiciness, and skin firmness, have a lower influence on the formation of the acceptability (*r* values of 0.76, 0.72, and 0.53, respectively).

Among the identified VOCs, 2-isobutylthiazole, 6-methyl-5-hepten-2-ol, (*E*)-2-hexenal, (*Z*)-3-hexen-1-ol, 2-phenylethyl alcohol, and benzyl alcohol can be considered as the most authentic volatile marker compounds for the cultivars, as they have high loading scores in the PC1 (Supplementary Table 9). A high loading score of 2-phenylethyl alcohol, which was measured in 6- to 13-fold higher concentrations in small-fruited tomato cultivars than in large-fruited cultivars (66), might indicate the contribution of this VOC to tomato aroma (16, 20). “Auriga” and “Green Zebra”, which differed from other cultivars by a higher loading score for sourness and color components (i.e., L*, b*, and h), were described by Chea et al. (32) as cultivars with high Mg concentrations in both leaves and fruits, which, in turn, correlated positively with TA content in fruits. Among the important VOCs in our study, three compounds correlated positively [(*E*)-2-hexenal, phenylethyl alcohol, and benzyl alcohol)] and two negatively (2-isobutylthiazole and 6-me-5-hepten-2-ol), with the acceptability of the cultivars (Table 5). These VOCs distinguish the cocktail cultivars (“Supersweet 100 F_1_” and “Goldita”) from the salad cultivars (“Roterno F_1_”, “Lyterno F_1_”, “Bocati F_1_”, and “Cappricia F_1_”). Overall, the results of the PCA suggest that the magnitude of the effect of each individual VOC is much smaller than the individual effect of other instrumental traits (especially TSS and TA) on the sample separation, highlighting the complexity of breeding for tomato flavor.

## Conclusion

Cultivar was the most important factor influencing the concentration of all measured instrumental and sensory traits. Results from the present work indicate that the main cultivar effect on VOCs and other traits was generally stronger than the cultivar and environmental condition (C × H × Y interaction) effect. The C × H × Y interaction had a significant effect on most color components, but not on TSS and TA. We observed 31 significant correlations between individual VOC and sensory attributes in tomato cultivars. The VOCs (*E*)-2-hexenal and phenylethyl alcohol were positively associated with sensory scores of sweetness, aroma, and acceptability. On the other hand, 2-isobutylthiazole and 6-methyl-5-hepten-2-ol were negatively correlated with TSS and the sensory parameters sweetness, aroma, and acceptability. This information may allow determining aroma compound composition distinctive of both within and between the cultivar groups, the diversity of the VOCs, as well as to identify cultivars with enhanced levels of the targeted traits. As a result of this study, organic breeders should use cultivars from a wide range of breeding programs to improve important quality and agronomic traits. As examples, salad tomatoes “Campari F_1_”, “Green Zebra”, and “Auriga” and cocktail tomatoes “Supersweet 100 F_1_”, “Sakura F_1_”, and “Black Cherry” showed important organoleptic attributes under organic low-input growing conditions. It remains a challenge for breeders and growers to reduce the trade-off of yield and quality.

## Data Availability Statement

The original contributions presented in this study are included in the article/Supplementary Material, further inquiries can be directed to the corresponding author.

## Author Contributions

CE, BH, and EP planned and designed the experimental setup. CE performed the experiments and analyzed the data. DU supervised the VOCs measurements and data analysis. CE, DU, MN, IS, BH, and EP wrote the manuscript. All authors read and approved the manuscript.

## Conflict of Interest

The authors declare that the research was conducted in the absence of any commercial or financial relationships that could be construed as a potential conflict of interest.

## Publisher’s Note

All claims expressed in this article are solely those of the authors and do not necessarily represent those of their affiliated organizations, or those of the publisher, the editors and the reviewers. Any product that may be evaluated in this article, or claim that may be made by its manufacturer, is not guaranteed or endorsed by the publisher.

## Footnotes

1
https://commonchemistry.cas.org/

2
http://www.fao.org/ag/agn/jecfa-flav/
