# Supplementary material for: Flavor and Other Quality Traits of Tomato Cultivars Bred for Diverse Production Systems as Revealed in Organic Low-Input Management
Source: Front Nutr. 2022 Jul 14;9:916642. doi: 10.3389/fnut.2022.916642 (PMC9331900; doi:10.3389/fnut.2022.916642)
Supplement: Supplementary file 2 [file Data_Sheet_1.PDF]

Supplementary Material

Table S1. Year of release, breeding background, average fruit weight, and usual production systems of 60 tomato cultivars used in Germany, Switzerland, and Austria (Chea et al., 2021).

Cultivar	Year of release	Breeder	Breeding background	Average fruit weight (g)	Production systems	IDX
Salad cultivars (>52g fruit $^{-1}$)						
Previa F_1	2011 [2]	Gautier	CON	173.1	a,c	2
Garance F_1	2015 [2]	Agri Obtentions	CON	154.5	e	5
Green Zebra	1972 [3]	Wagner	ORG	153	c	3
Diplom F_1	1989 [4]	Hild	CON	136.8	b	2
Cappricia F_1	2009 [2]	Rijk Zwaan	CON	131.5	g	7
Rougella F_1	1999 [2]	Rijk Zwaan	CON	126.4	c	3
Sparta F_1	1994 [5]	Enza	CON	125.8	e	5
Bocati F_1	2011 [2]	Enza	CON	124.4	c,d	3.5
Phantasia F_1	2006 [2]	De Ruiter	CON	122.7	a,b	1.5
Mecano F_1	2004 [2]	Rijk Zwaan	CON	122.2	e,g	6
Hamlet F_1	2009 [2]	Nunhems	CON	120.4	d	4
Lyterno F_1	2010 [2]	Rijk Zwaan	CON	115.9	e,f,g	6
Nordica F_1	2014 [2]	Enza	CON	115.5	c,d,e	4
Moneymaker	1972 [2]	Hild	CON	113.8	a,c	2
Pannovy F_1	1991 [5]	Syngenta	CON	107.6	c,e	4
Roterno F_1	2007 [2]	Rijk Zwaan	CON	106.7	d,e,f	5
Hildares F_1	1978 [2]	Hild	CON	99	b	2
Bonner Beste	1955 [4]	Reinhold	ND	95.2	*	*
Tica	2011 [2]	Kultursaat	ORG	94.2	c,e	4
Ricca	2015 [6]	Reinsaat	ORG	92.1	e	5
Aroma	2015 [7]	Kultursaat	ORG	87.1	c	3
Rheinlands Ruhm	1945 [8]	Unknown	ND	85	b	2
Lukullus	1956 [4]	Reinhold	ND	83.9	*	*

Goldene Königin	1882	[14]	Unknown	ND	76.6	c,d	3.5
Harzfeuer F_1	1959	[4]	IZQ	CON	76.4	a,b,c,d	2.5
Auriga	1980	[4]	Saatzucht Quedlinburg	CON	71.5	c,e	4
Haubners Vollendung	1950	[8]	Unknown	ND	70.3	*	*
Dorenia	2012	[2]	Kultursaat	ORG	68.2	a	1
Roi Humbert Jaune	1898	[9]	Unknown	ND	64.9	c	3
Hellfrucht	1955	[4]	Fetzer	ND	64.3	*	*
Campari F_1	1996	[2]	Enza	CON	63.3	e,f	5.5
Matina	1978	[2]	Hild	CON	55.2	a,b,c,d	2.5
Black Plum	1998	[10]	Unknown	ND	52.2	a,c	2
Cocktail cultivars (<52 g fruit^{-1})							
Amoroso F_1	2005	[2]	Rijk Zwaan	CON	50.8	f,g	6.5
Annamay F_1	2010	[2]	Enza	CON	46	e	5
Quedlinburger Frühe Liebe	1951	[8]	Unknown	ND	43.4	a	1
Ruthje	2008	[4]	Kultursaat	ORG	42.3	c,e	4
König Humbert	1880	[11]	Unknown	ND	37.7	*	*
Clou	2010	[2]	OOTP	ORG	34.4	a,b	1.5
Tastery F_1	2011	[2]	Rijk Zwaan	CON	33.5	d,e,f,g	5.5
Primabella	2012	[2]	OOTP	ORG	28.1	a,c	2
Sakura F_1	1999	[2]	Enza	CON	23.7	c,d,e,f	4.5
Black Cherry	2009	[2]	Reinsaat	ND	23	c,d	3.5
Cerise Gelb	2005	[12]	OOTP	ND	22.9	a,b	1.5
Yellow Submarine	2002	[2]	Unknown	ND	22.2	c	3
Zuckertraube	1994	[2]	Reinsaat	ND	21.9	a,b,c	2
Dorada	2010	[2]	OOTP	ORG	21.3	a,b	1.5
Primavera	2010	[2]	OOTP	ORG	21.3	a,b	1.5
Philovita F_1	2007	[2]	De Ruiter	CON	19.4	a,b,c	2
Trixi	2014	[4]	Kultursaat	ORG	19.3	c,e	4
Trilly F_1	2006	[2]	ISI Sementi	CON	19.3	d	4
Benarys Gartenfreude1	1950	[4]	Benary	CON	18.5	*	*
Bartelly F_1	2014	[2]	De Bolster	ORG	18.4	c,e	4

Golden Pearl F_1	2008	2	Hild	CON	18.4	d	4
Resi	2010	2	OOTP	ND	17.3	a,b	1.5
Supersweet 100 F_1	1992	2	Syngenta	CON	15.7	a,b,c,d,	2.5
Goldita	1997	8	De Ruiter/Arche Noah	CON	15.6	c	3
Sliwowidnij	2012	13	Unknown	ND	9.6	C	3
Rote Murmel	1995	13	Unknown	ND	5.7	a,b	1.5
Golden Currant	1975	13	Unknown	ND	5.4	a,b	1.5

Cultivars shown in bold are the 20 cultivars selected from 2015 for further evaluation in 2016. Year of release in italic are not known with certainty.
IZQ = Institut für Züchtungsforschung Quedlinburg; OOTP = Organic Outdoor Tomato Project (www.uni-kassel.de/go/freilandtomatenprojekt, accessed on 01 August 2021).

[1] Syn. Freude, syn. Gardener's Delight; [2] European Commission [1]; [3] T. Wagner (2016) pers. comm.; [4] Bundessortenamt (2016) pers. comm. (www.bundessortenamt.de); [5] Bundessortenamt [2]; [6] Reinsaat (2015) pers. comm. (www.reinsaat.at)*;* [7] S. Wedemeyer/Kultursaat e.V. (2016) pers. comm. (www.kultursaat.org); [8] Arche Noah (2015) pers. comm. (www.arche-noah.at); [9] Haage and Schmidt [3]; [10] ProSpecieRara (2016) pers. comm. (www.prospecierara.de); [11] Munro [4]; [12] Dreschflegel [5]; [13] Culinaris (2015) pers. comm. (www.culinaris-saatgut.de); [14] Livin.gston and Smith [6]

Breeding background: CON=Conventional, OR =Organic, ND=Not documented.
Weight per fruit=average weight per fruit (g) derived from the experiment 2015.

Suitable organic low of the cultivars: a=organic outdoor, b=conventional outdoor, c= extensive organic indoor, d=extensive conventional indoor, e=intensive organic indoor, f=intensive conventional indoor, g=hydroponic, *=hardly grown anymore. This information was collected with extension services, research stations, breeders, seed companies, and the IPK Genebank. The intensity index (IDX) was calculated as the average value of suitable growing systems with a=1, b=2, c=3, d=4, e=5, f=6, g=7. Thus, the lowest IDX corresponds to the lowest input level. The IDX is not proportional to CO_2 equivalents per unit tomato fruits.

Reference: Chea, L., Erika, C., Naumann, M., Smit, I., Horneburg, B., Pawelzik, E., 2021. Morphological, leaf Nutrient, and fruit quality characteristics of diverse tomato cultivars under organic low-input management. Sustainability 13, 12326. https://doi.org/10.3390/su132112326

Table S2. VOCs identified across all samples

CAS number	IUPAC name	Abbreviation	Identification
100-52-7	benzaldehyde	benzald	2
100-51-6	benzyl alcohol	benzylalc	2
5392-40-5	citral	citral	2
432-25-7	β-cyclocitral	cyclocit	1
23696-85-7	β-damascenone	damasc	2
25152-84-5	decadienal	deca	2
104-76-7	2-ethyl-1-hexanol	ethexanol	2
97-53-0	eugenol	eugenol	2
1117-52-8	farnesylacetone	farnesylac	1
3796-70-1	(*E*)-geranylacetone	gera	1
141-27-5	geranial	geranial	2
66-25-1	hexanal	hexanal	2
111-27-3	1-hexanol	hexanol	2
6728-26-3	(*E*)-2-hexenal	hexenal	2
928-96-1	(*Z*)-3-hexen-1-ol	hexenol	2
14901-07-6	β-ionone	ionone	2
110-27-0	isopropylmyristate	iprop	2
18640-74-9	2-isobutylthiazole	isobut	2
78-70-6	linalool	linalool	2
624-41-9	2-methylbutylacetate	mebuOAc	2
503-74-2	3-methylbutanoic acid	mebutacid	2
-	methylheptadione	meheptdione	1
1569-60-4	6-methyl-5-hepten-2-ol	meheptenol	2
110-93-0	6-methyl-5-hepten-2-one	meheptone	2
119-36-8	methylsalicylate	meOSal	2
79-31-2	2-methylpropanoic acid	mepropacid	2
112-05-0	nonanoic acid	nonacid	2
124-07-2	octanoic acid	octacid	2
124-13-0	octanal	octanal	2
60-12-8	phenylethyl alcohol	phenylet	2
98-55-5	α-terpineol	terpineol	2

CAS: Chemical Abstracts Service (a numeric identifier of the chemical compounds); IUPAC: International Union of Pure and Applied Chemistry; Identification code: 1- tentatively identified by MS library search (NIST 14, Wiley, Nbs75k); 2- fully identified by MS library search and co-elution of authentic reference substances

Table S3. Concentration of VOCs of 33 salad cultivars grown in 2015 (norm-%)

Cultivar	hexanal	(*E*)-2-hexenal	octanal	6-methyl-5-heptene-2-one	1-hexanol	(*Z*)-3-hexen-1-ol	2-isobutylthiazole	2-ethyl-1-hexanol	benzaldehyde	linalool	methylheptadione	β-cyclocitral	3-mebutanoic acid	α-terpineol	geranial	decadienal	β-damascenone	(*E*)-geranylacetone	2-mepropanoic acid	benzylalcohol	phenylethylalcohol	β-ionone	octanoic acid	nonanoic acid
Previa F1	26.5	12.2	5.1	18.2	1.7	3.6	9.4	1.0	0.2	2.2	0.0	1.1	0.5	0.1	2.5	0.0	2.2	9.9	0.4	0.0	1.3	1.3	0.5	0.0
Garance F1	33.9	7.8	2.6	24.4	1.4	1.9	12.6	0.7	0.0	1.6	0.0	0.6	0.7	0.2	1.7	0.3	1.5	6.1	0.1	0.0	1.2	0.7	0.0	0.0
Green Zebra	27.2	14.1	3.9	23.9	2.0	2.4	9.8	0.9	0.3	3.3	0.0	0.1	0.5	0.2	0.0	0.0	8.4	1.5	0.0	0.0	0.2	0.0	0.3	1.0
Diplom F1	24.9	4.0	6.7	19.8	3.6	4.5	14.6	1.0	0.0	3.1	0.0	1.3	0.0	0.7	1.9	0.0	2.2	6.8	0.0	0.0	0.0	1.2	0.1	3.8
Cappricia F1	27.1	6.8	7.0	19.1	0.8	3.0	9.9	1.3	0.0	4.0	0.0	2.0	0.3	0.3	2.3	0.0	5.0	8.2	0.2	0.0	0.4	1.9	0.4	0.0
Rougella F1	38.6	13.8	4.4	16.2	1.6	2.8	3.8	0.7	0.2	2.5	0.1	0.9	0.5	0.3	1.6	0.0	3.0	6.9	0.0	0.0	0.8	1.0	0.2	0.0
Sparta F1	35.4	11.7	4.1	13.3	1.7	2.4	9.6	0.7	0.1	3.7	0.0	1.1	0.3	0.4	1.3	0.0	4.1	7.5	0.4	0.0	0.3	1.1	0.1	0.7
Bocati F1	25.6	6.8	4.4	19.3	2.6	3.3	15.9	0.8	0.2	4.8	0.0	1.2	0.4	0.1	1.4	0.0	4.0	7.0	0.1	0.0	0.2	1.1	0.0	0.7
Phantasia F1	20.3	11.2	7.4	14.4	1.4	6.9	9.0	2.5	0.8	4.6	0.0	1.3	0.0	0.0	1.9	0.0	7.5	8.1	0.3	0.0	0.0	1.8	0.6	0.0
Mecano F1	23.8	7.9	6.9	16.9	1.5	4.1	9.4	1.0	0.4	5.3	0.0	1.8	0.2	0.8	2.7	0.0	5.4	9.0	0.1	0.0	0.1	1.9	0.8	0.0
Hamlet F1	40.6	12.5	4.5	13.9	1.3	2.0	7.1	0.8	0.1	2.1	0.0	0.8	0.3	0.4	1.9	0.0	2.4	7.6	0.2	0.0	0.3	0.9	0.3	0.0
Lyterno F1	16.5	5.1	6.6	23.3	1.0	3.6	14.6	1.2	0.2	4.6	0.0	2.3	0.4	0.1	3.2	0.0	5.5	8.2	0.4	0.0	0.3	2.1	0.8	0.0
Nordica F1	27.4	11.1	4.6	18.0	1.0	2.3	13.1	1.1	0.1	4.0	0.0	1.2	0.2	0.5	1.8	0.0	3.2	8.8	0.1	0.0	0.1	1.1	0.2	0.0
Moneymaker	29.3	8.1	5.7	17.6	2.6	3.4	10.9	1.4	0.0	2.4	0.0	1.3	0.1	0.3	2.6	0.0	1.5	8.1	0.2	0.0	0.0	1.4	0.2	2.8
Pannovy F1	28.5	13.8	4.4	18.8	1.9	2.8	8.8	0.7	0.3	4.1	0.0	1.0	0.7	0.4	1.5	0.0	4.8	6.4	0.0	0.0	0.0	1.0	0.0	0.0
Roterno F1	24.3	4.6	5.1	26.1	1.4	2.4	16.4	0.8	0.3	2.8	0.1	1.4	0.5	0.1	2.0	0.0	3.2	6.3	0.3	0.0	0.3	1.3	0.3	0.0
Hildares F1	22.8	10.4	3.3	19.7	2.7	4.5	9.2	1.2	0.3	5.7	0.0	1.2	0.2	0.5	1.8	0.0	4.7	7.6	0.0	0.0	0.0	1.5	0.1	2.5
Bonner Beste	14.2	10.7	7.7	24.2	1.9	5.4	10.9	1.5	0.1	3.1	0.0	1.5	0.3	0.0	3.1	0.0	3.0	7.3	0.7	0.0	0.0	1.7	0.8	1.9
Tica	28.3	8.6	5.6	19.4	1.7	2.8	9.9	0.8	0.2	2.0	0.0	1.4	0.5	0.6	3.0	0.1	1.9	9.4	0.4	0.0	1.0	1.7	0.7	0.3
Ricca	28.3	12.9	6.2	16.7	1.7	3.0	9.5	0.9	0.2	2.6	0.2	1.5	0.1	0.8	1.9	0.0	3.8	7.1	0.2	0.0	0.5	1.5	0.2	0.3
Aroma	27.8	10.9	3.6	19.7	3.0	3.5	12.5	0.7	0.1	2.9	0.0	1.2	0.4	0.2	1.2	0.1	4.5	5.4	0.0	0.0	0.6	1.0	0.1	0.7
Rheinlands Ruhm	24.3	9.8	5.9	16.8	3.0	4.3	10.5	1.1	0.5	6.1	0.0	1.8	0.1	0.1	1.6	0.0	4.1	6.9	0.1	0.0	0.0	1.4	0.3	1.2
Lukullus	25.8	6.0	6.2	18.4	3.6	5.3	14.2	1.3	0.1	3.6	0.0	1.6	0.1	0.0	1.9	0.0	2.2	5.6	0.3	0.0	0.2	1.3	0.3	2.1
Goldene Königin	28.7	16.2	5.0	12.0	1.9	4.1	17.2	1.2	0.4	2.7	0.0	0.0	0.7	0.2	0.0	0.2	4.3	0.6	0.3	0.0	0.0	0.0	0.8	3.6
Harzfeuer F1	24.0	8.8	5.7	21.8	3.2	4.8	11.0	0.7	0.4	2.8	0.1	1.3	0.6	0.2	2.3	0.0	2.9	6.5	0.1	0.0	0.1	1.1	0.9	0.8
Auriga	16.7	12.4	4.2	14.1	1.8	4.6	12.0	0.8	0.6	3.6	0.0	7.0	1.0	0.0	0.2	0.0	9.1	3.4	0.6	0.0	0.6	5.7	1.0	0.8
Haubners Vollendung	19.6	9.3	4.4	25.3	1.7	3.4	15.1	0.6	0.0	4.5	0.0	1.3	0.2	0.1	1.5	0.0	4.2	6.0	0.0	0.0	0.0	1.1	0.4	1.2
Dorenia	34.7	7.6	4.8	21.4	1.9	2.8	10.3	0.9	0.2	2.1	0.0	0.9	0.9	0.1	1.6	0.1	1.9	6.0	0.1	0.0	0.1	0.8	0.0	0.8
Roi Humbert Jaune	37.1	21.5	5.4	4.0	2.2	5.0	10.5	1.1	0.0	4.6	0.0	0.0	0.4	0.2	0.0	0.0	3.9	0.8	0.4	0.0	0.0	0.0	0.3	2.6
Hellfrucht	23.2	11.7	4.7	17.5	2.9	5.3	8.1	1.0	0.3	5.4	0.0	1.7	0.3	0.3	1.7	0.0	5.8	6.2	0.3	0.0	0.0	1.5	0.2	1.9
Campari F1	18.2	12.3	6.9	18.4	2.5	4.8	15.4	0.9	0.3	2.2	0.2	1.3	0.4	1.3	1.9	0.0	4.5	5.2	0.0	0.0	1.0	1.2	0.7	0.4
Matina	28.6	11.3	4.9	18.6	3.0	3.6	12.6	0.9	0.1	2.4	0.0	1.0	0.2	0.3	1.8	0.1	3.4	5.8	0.2	0.0	0.0	1.0	0.0	0.3
Black Plum	45.3	8.0	4.3	15.5	2.9	2.7	8.2	1.0	0.1	1.3	0.0	0.8	0.7	0.2	1.8	0.1	0.8	4.5	0.0	0.0	0.1	0.8	0.1	0.7
Mean	27.2	10.3	5.2	18.4	2.1	3.7	11.3	1.0	0.2	3.4	0.0	1.4	0.4	0.3	1.7	0.0	3.9	6.4	0.2	0.0	0.3	1.3	0.4	0.9
SD	7.0	3.6	1.2	4.4	0.8	1.2	3.0	0.4	0.2	1.2	0.0	1.1	0.2	0.3	0.8	0.1	1.9	2.2	0.2	0.0	0.4	0.9	0.3	1.1
HSD (0.05)	16.7	5.7	3.1	8.2	2.2	3.1	5.6	1.7	0.6	3.3	0.2	0.9	1.0	1.1	1.3	0.3	3.4	3.0	0.9	0.0	0.4	0.8	0.8	1.6
Cultivar (C)	***	***	***	***	***	***	***	ns	***	***	ns	***	**	**	***	**	***	***	ns	ND	***	***	***	***
Harvest (H)	***	***	ns	***	***	**	*	***	ns	***	ns	***	***	***	***	**	ns	ns	***	ND	***	***	ns	ns
Interaction (C x H)	ns	***	*	***	ns	**	***	ns	ns	*	ns	ns	ns	**	**	ns	***	***	ns	ND	***	***	ns	**

Mean values are given for each of the 33 salad cultivars as mean from samples grown in 2015; SD=standard deviation; ns indicates a nonsignificant difference; *, ** and *** indicate significance differences of each factor and interaction at p<0.05, p<0.01 and p <0.001, respectively; HSD (0.05)=critical value for comparisons by Tukey’s honestly significant difference (HSD) tests at p<0.05. Cultivar name “Goldene Koenigin” is written as “Goldene Königin”. The cultivars are arranged in descending order according to their average fruit weight

Table S4. Concentration of VOCs of 27 cocktail cultivars grown in 2015 (norm-%)

Cultivar	hexanal	(*E*)-2-hexenal	octanal	6-methyl-5-heptene-2-one	1-hexanol	(*Z*)-3-hexen-1-ol	2-isobutyl-thiazole	2-ethyl-1-hexanol	benzal-dehyde	linalool	methyl-heptadi-one	β-cyclo-citral	3-mebuta-noic acid	α-terpineol	gera nial	decadi enal	β-damas-cenone	(*E*)-geranyl-acetone	2-mepro-panoic acid	benzyl-alcohol	phenyl-ethyl-alcohol	β-ionone	octa-nioc acid	nona-noic acid
Amoroso F1	27.3	9.0	6.1	19.4	1.9	3.6	7.5	1.2	0.4	2.8	0.0	1.4	0.4	0.4	1.6	0.1	5.5	7.1	0.4	0.0	1.6	1.5	0.7	0.0
Annamay F1	21.5	11.0	4.0	18.6	2.3	4.4	14.9	0.8	0.1	2.4	0.1	1.3	0.2	0.2	1.1	0.0	9.2	5.4	0.3	0.0	0.8	1.1	0.3	0.0
Quedlinburger Frühe Liebe	28.1	10.1	4.1	16.7	2.4	2.8	14.1	0.6	0.3	4.0	0.2	1.0	0.1	0.1	1.2	0.0	5.0	6.3	0.2	0.4	0.9	0.9	0.1	0.3
Ruthje	24.4	10.2	5.5	19.9	2.1	3.2	11.5	0.9	0.4	0.8	0.0	1.6	0.4	0.3	3.3	0.0	1.1	9.3	0.5	0.2	2.3	1.7	0.5	0.0
König Humbert	48.5	11.2	2.8	16.1	1.2	1.5	5.5	0.4	0.2	0.9	0.0	0.9	1.2	0.1	1.0	0.0	2.4	4.2	0.2	0.0	0.1	0.8	0.3	0.4
Clou	23.5	17.1	5.3	13.9	2.7	4.0	25.5	1.2	0.2	1.8	0.0	0.0	0.6	0.0	0.0	0.0	3.2	0.7	0.2	0.0	0.1	0.1	0.1	0.0
Tastery F1	26.4	12.7	6.0	15.6	1.7	5.5	6.1	1.6	0.0	1.9	0.0	3.2	0.0	0.0	2.6	0.0	4.2	8.2	0.4	0.0	0.5	3.2	0.1	0.0
Primabella	38.7	14.8	2.4	11.3	1.8	3.2	10.4	0.6	0.0	0.8	0.0	1.2	0.7	0.1	1.2	0.0	4.6	4.9	0.1	0.0	1.2	0.9	0.1	1.0
Sakura F1	17.7	16.2	5.0	18.2	1.9	3.7	14.9	1.1	0.3	2.2	0.0	1.4	0.2	0.0	1.6	0.0	6.7	4.5	0.3	0.4	2.0	1.2	0.4	0.0
Black Cherry	40.7	11.8	4.9	11.4	2.5	3.3	7.2	0.9	0.1	4.5	0.0	1.2	0.4	0.8	1.4	0.0	4.0	3.0	0.5	0.1	0.1	0.8	0.4	0.0
Cerise gelb	29.6	16.1	3.2	22.8	1.6	3.3	14.0	0.6	0.3	0.6	0.0	0.1	0.8	0.1	0.0	0.0	5.8	0.2	0.0	0.0	0.5	0.1	0.1	0.1
Yellow Submarine	31.5	14.5	3.0	19.9	1.6	2.8	16.3	0.7	0.6	2.1	0.0	0.0	1.0	0.1	0.0	0.0	3.2	0.4	0.1	0.0	0.6	0.0	0.6	1.1
Zuckertraube	22.6	15.0	6.3	17.8	2.6	5.7	2.8	1.8	0.2	2.7	0.1	2.0	0.0	0.0	2.2	0.0	6.3	5.7	0.4	1.7	2.2	1.8	0.0	0.0
Dorada	34.4	24.9	4.6	5.3	2.4	6.3	11.5	1.2	0.3	1.6	0.0	0.0	0.1	0.0	0.0	0.0	5.6	0.6	0.5	0.0	0.2	0.0	0.6	0.0
Primavera	34.6	13.1	5.0	10.6	4.1	6.0	9.9	1.4	0.1	0.3	0.0	2.3	0.1	1.7	0.9	0.0	3.0	4.3	0.3	0.0	0.0	1.9	0.3	0.0
Philovita F1	48.1	11.7	3.2	11.9	2.5	2.7	2.5	0.4	0.1	0.5	0.0	1.3	0.6	0.2	1.8	0.1	2.2	6.3	0.2	0.0	2.4	1.1	0.3	0.0
Trixi	28.8	15.8	5.2	18.3	2.8	5.2	3.5	0.9	0.1	1.8	0.0	2.1	0.2	0.1	1.4	0.0	4.7	5.5	0.3	0.4	1.4	1.8	0.0	0.0
Trilly F1	40.0	12.3	4.5	11.8	2.8	4.5	7.1	0.9	0.1	2.3	0.0	1.2	0.5	0.0	1.4	0.1	4.0	3.9	0.1	0.0	1.5	0.8	0.2	0.0
Benarys Gartenfreude	34.7	9.3	4.9	27.1	2.4	4.9	3.7	0.6	0.1	0.5	0.0	1.0	0.2	0.6	2.1	0.5	1.4	4.2	0.1	0.0	0.2	1.0	0.5	0.0
Bartelly F1	32.8	16.3	3.7	13.3	1.2	3.1	8.8	0.8	0.3	1.3	0.2	1.5	0.5	0.1	1.3	0.1	4.8	5.2	0.6	0.0	2.2	1.3	0.6	0.0
Golden Pearl F1	36.5	18.2	3.3	4.1	2.0	5.4	15.1	0.8	0.2	3.5	0.1	0.3	0.3	0.0	0.0	0.0	4.9	0.2	0.7	0.1	2.9	0.0	1.0	0.5
Resi	31.7	8.0	4.9	23.5	3.3	3.9	10.0	0.7	0.1	0.8	0.0	0.9	0.6	0.3	1.7	0.0	4.2	4.1	0.0	0.0	0.2	0.8	0.1	0.0
Supersweet 100 F1	20.9	15.0	5.6	18.9	1.7	5.3	3.7	0.9	0.2	5.8	0.0	1.8	0.3	0.1	1.6	0.0	7.2	5.2	0.4	0.3	3.2	1.1	0.7	0.0
Goldita	22.1	16.3	5.7	12.4	2.3	4.9	2.9	1.2	0.0	2.0	0.0	0.7	0.0	0.0	0.6	0.0	5.2	18.6	0.2	1.0	3.1	0.6	0.1	0.0
Sliwowidnij	22.7	14.4	3.6	19.7	1.7	3.9	14.1	0.8	0.1	4.8	0.0	0.0	0.5	0.1	0.0	0.0	12.2	0.2	0.5	0.0	0.0	0.0	0.3	0.4
Rote Murmel	34.5	7.4	3.9	23.7	3.9	6.0	5.3	1.0	0.3	1.0	0.0	1.3	0.9	1.3	1.2	0.0	1.7	3.8	0.0	0.4	1.1	1.0	0.3	0.0
Golden Currant	27.9	7.1	2.5	14.5	5.8	7.3	17.5	0.9	1.2	1.2	0.0	0.1	0.6	1.6	0.2	0.0	1.3	0.7	0.1	0.0	9.0	0.0	0.4	0.0
Mean	30.8	13.3	4.4	16.1	2.4	4.3	9.9	0.9	0.2	2.0	0.0	1.1	0.4	0.3	1.2	0.0	4.6	4.5	0.3	0.2	1.5	0.9	0.3	0.1
SD	8.0	3.9	1.2	5.4	1.0	1.4	5.6	0.3	0.2	1.4	0.1	0.8	0.3	0.5	0.9	0.1	2.5	3.8	0.2	0.4	1.8	0.7	0.3	0.3
HSD (0.05)	13.1	9.2	2.4	8.4	2.0	3.0	4.8	1.5	0.6	1.4	0.3	0.7	0.8	2.9	1.2	0.2	2.8	3.3	1.2	0.3	0.8	0.7	0.6	0.5
Cultivar (C)	***	***	***	***	***	***	***	*	***	***	*	***	***	ns	***	***	***	***	ns	***	***	***	***	***
Harvest (H)	***	***	**	***	ns	***	***	*	ns	***	**	***	*	**	**	***	ns	ns	***	***	***	ns	ns	*
Interaction (C x H)	***	***	*	***	***	***	***	ns	ns	***	*	***	***	ns	*	***	***	***	ns	***	***	**	ns	***

Mean values are given for each of the 27 cocktail cultivars as mean from samples grown in 2015; SD=standard deviation; ns indicates a nonsignificant difference; *, ** and *** indicate significance differences of each factor and interaction at p<0.05, p<0.01 and p <0.001, respectively; HSD (0.05) = critical value for comparisons by Tukey's honestly significant difference (HSD) tests at p<0.05. Cultivar names "Quedlinburger Fruehe Liebe" and "Koenig Humbert" is written as "Quedlinburger Frühe Liebe", and "König Humbert", respectively. The cultivars are arranged in descending order according to the average fruit weight

Table S5. ANOVA of concentration of VOCs (norm-%) in the 20 tomato cultivars grown in 2015 and 2016 as well as comparison between its fruit type with the student t-test for significance

VOCs	ANOVA, source of variation						Concentration (mean ± SD)					Student's t-test[a]
	Cultivar, C	Harvest, H	Year, Y	C × H	C × Y	C × H × Y	2015 n=120	2016 n=120	Over two years n=240	Cocktail cultivar n=144	Salad cultivar n=96	
hexanal[a,b]	***	***	***	ns	***	ns	26.4±11.6	44.0±9.36	35.2±13.7	36.5±12.9	33.3±14.7	ns
2-methylbutylacetate[a,b]	***	ns	***	ns	***	ns	0.00±0.00	0.59±2.57	0.29±1.83	0.40±2.17	0.14±1.17	ns
(*E*)-2-hexenal[a,b]	***	***	***	***	***	***	11.2±6.53	6.36±2.95	8.78±5.61	9.49±6.30	7.74±4.21	*
octanal[a,b]	***	**	***	***	***	ns	5.09±1.62	2.30±1.10	3.70±1.96	3.63±1.79	3.79±2.20	ns
6-methyl-5-heptene-2-one[a,b]	***	***	***	***	***	*	18.3±7.33	16.5±6.99	17.4±7.21	16.84±7.08	18.2±7.34	ns
1-hexanol[a,b]	***	ns	***	***	***	*	2.12±1.22	1.16±0.66	1.64±1.09	1.75±1.02	1.49±1.17	ns
(*Z*)-3-hexen-1-ol[a,b]	***	***	***	***	ns	**	4.02±1.87	1.37±0.93	2.69±1.98	2.89±2.10	2.39±1.77	ns
2-isobutylthiazole[a,b]	***	***	***	***	**	ns	10.3±4.87	11.9±6.17	11.1±5.60	9.72±5.70	13.1±4.80	***
6-methyl-5-hepten-2-ol[a,b]	***	*	***	**	***	**	0.00±0.00	0.41±0.38	0.20±0.34	0.15±0.28	0.28±0.40	**
2-ethyl-1-hexanol[a,b]	***	ns	***	ns	ns	ns	0.96±0.61	0.70±0.29	0.83±0.49	0.84±0.51	0.81±0.48	ns
benzaldehyde[a,b]	**	ns	***	ns	**	ns	0.20±0.28	0.00±0.00	0.10±0.22	0.08±0.18	0.14±0.27	*
linalool[a,b]	***	***	***	ns	***	**	2.68±1.82	1.34±1.08	2.01±1.64	1.64±1.55	2.56±1.62	***
methylheptadione[a]	*	**	***	*	*	*	0.04±0.13	0.00±0.00	0.02±0.09	0.01±0.08	0.03±0.11	ns
β-cyclocitral[a]	***	***	***	ns	***	ns	1.72±1.47	1.30±0.91	1.51±1.23	1.43±0.82	1.63±1.66	ns
3-mebutanoic acid[a,b]	***	***	***	ns	***	*	0.40±0.48	0.10±0.21	0.25±0.40	0.19±0.34	0.33±0.47	*
α-terpineol[a,b]	ns	***	ns	ns	ns	ns	0.33±1.11	0.19±0.28	0.26±0.81	0.26±0.89	0.27±0.67	ns
geranial[a,b]	***	ns	***	**	***	**	1.55±0.95	0.00±0.00	0.78±1.03	0.74±0.90	0.83±1.19	ns
citral[a,b]	***	ns	***	ns	***	ns	0.00±0.00	1.78±1.14	0.89±1.20	0.98±1.28	0.76±1.06	ns
decadienal[a,b]	***	***	***	***	***	***	0.03±0.17	0.00±0.00	0.02±0.12	0.03±0.15	0.00±0.00	ns
methylsalicylate[a,b]	***	***	***	***	***	***	0.00±0.00	1.15±1.66	0.58±1.30	0.53±1.25	0.64±1.39	ns
β-damascenone[a,b]	***	**	***	***	***	***	5.15±2.75	1.41±1.75	3.28±2.96	3.19±2.84	3.41±3.15	ns
(*E*)-geranylacetone[a]	***	ns	***	***	***	***	6.03±4.11	4.63±4.33	5.33±4.27	5.46±4.34	5.14±4.17	ns
2-mepropanoic acid[a,b]	*	**	ns	ns	ns	ns	0.26±0.51	0.24±0.30	0.25±0.42	0.26±0.44	0.23±0.38	ns
benzylalcohol[a,b]	***	ns	***	*	***	***	0.10±0.30	0.23±0.44	0.16±0.38	0.22±0.44	0.08±0.24	**
phenylethylalcohol[a,b]	***	***	***	***	***	***	0.92±1.19	0.79±0.97	0.86±1.09	1.12±1.27	0.46±0.54	***
β-ionone[a,b]	***	*	***	***	***	***	1.47±1.22	1.09±0.66	1.28±1.00	1.21±0.66	1.39±1.34	ns
eugenol[a,b]	***	ns	***	ns	***	ns	0.00±0.00	0.18±0.22	0.09±0.18	0.07±0.16	0.12±0.21	*
farnesylacetone[a]	***	*	***	ns	***	ns	0.00±0.00	0.29±0.39	0.14±0.31	0.13±0.31	0.17±0.32	ns
isopropylmyristate[a,b]	-	-	-	-	-	-	Nd	Nd	Nd	Nd	Nd	-
octanoic acid[a,b]	***	ns	***	ns	***	ns	0.44±0.41	0.00±0.00	0.22±0.36	0.18±0.29	0.27±0.44	ns
nonanoic acid[a,b]	***	ns	***	***	***	***	0.24±0.50	0.00±0.00	0.12±0.38	0.04±0.25	0.23±0.48	***

[a]tentatively identified by MS library search (NIST, Wiley, Nbs75k); [b]fully identified by co-elution with authentic reference substances and GC-MS spectra. Data are normalized to the total relative level of identified VOCs (norm-%); each mean represents six biological replicates (over two years)

and three biological replicates (within one year). ANOVA: analysis of variance; n=number of observations, SD: standard deviation; Nd=not detectable

Table S6: see Excel file

Table S7. Correlation coefficients, between VOCs in the 20 cultivars over two harvest dates and two years, n=240

**VOCs**	**(*E*)-2-hexenal**	**octanal**	**6-methyl-5-hepten-2-one**	**1-hexanol**	**(*Z*)-3-hexen-1-ol**	**6-methyl-5-hepten-2-ol**	**2-ethyl-1-hexanol**	**benzaldehyde**	**β-cyclocitral**
(*E*)-2-hexenal	1.00								
octanal	-0.06	1.00							
6-methyl-5-hepten-2-one	-0.22	-0.03	1.00						
1-hexanol	0.03	0.01	0.06	1.00					
(*Z*)-3-hexen1-ol	0.39	0.30	0.00	0.69**	1.00				
6-methyl-5-hepten-2-ol	-0.40	0.19	-0.18	-0.44	-0.50*	1.00			
2-ethyl-1-hexanol	0.22	0.69**	-0.45*	0.00	0.27	-0.04	1.00		
benzaldehyde	0.12	-0.10	0.08	-0.12	-0.08	-0.10	-0.24	1.00	
β-cyclocitral	0.19	0.05	-0.34	0.03	0.42	-0.32	0.20	0.40	1.00
3-methylbutanoic acid	-0.23	-0.57**	0.22	-0.17	-0.45*	0.09	-0.66**	0.38	0.04
geranial	-0.34	0.62**	0.07	-0.27	-0.12	0.59**	0.21	-0.20	-0.21
methylsalicylate	-0.07	0.00	-0.28	0.09	0.09	0.01	0.15	-0.10	0.06
geranial	0.04	0.28	-0.18	0.11	0.25	0.12	0.43	-0.37	-0.17
2-methylpropanoic acid	0.26	0.34	-0.54*	-0.14	0.15	-0.14	0.55*	0.42	0.63**
benzyl alcohol	0.53*	0.02	-0.10	0.07	0.30	-0.17	0.25	-0.14	-0.10
phenylethyl alcohol	0.62**	0.06	-0.05	-0.16	0.25	-0.22	0.16	-0.02	-0.07
β-ionone	0.13	0.16	-0.38	0.05	0.45*	-0.30	0.32	0.31	0.98**
eugenol	-0.16	0.10	-0.53*	-0.15	-0.14	0.26	0.16	-0.05	0.07
farnesylacetone	0.10	-0.22	0.01	0.07	0.36	-0.20	-0.21	0.33	0.71**
octanoic acid	0.17	0.34	0.12	-0.19	0.16	0.04	0.01	0.72**	0.47*

nonanoic acid	0.07	-0.62*	-0.10	-0.03	-0.14	0.06	-0.45*	0.37	0.12

Data used for the Pearson correlation are derived from each of the 20 cultivars as mean from both years. Significant correlation is indicated by asterisks: *p≤0.05 and **p≤0.01. The compounds which showed no significant correlations with other VOCs were not included in the table.

Table S7. Continue

**VOCs**	**3-methyl-butanoic acid**	**geranial**	**methyl-salicylate**	**geranial**	**2-methyl-propanoic acid**	**benzyl alcohol**	**phenylethyl alcohol**	**β-ionone**	**eugenol**	**farnesyl acetone**
3-methylbutanoic acid	1.00									
geranial	-0.22	1.00								
methylsalicylate	-0.08	0.19	1.00							
geranial	-0.50*	-0.01	-0.03	1.00						
2-methylpropanoic acid	-0.29	-0.03	0.16	-0.05	1.00					
benzyl alcohol	-0.39	-0.30	-0.28	0.73**	0.07	1.00				
phenylethyl alcohol	-0.41	-0.20	-0.29	0.54*	0.05	0.83**	1.00			
β-ionone	-0.09	-0.11	0.11	-0.07	0.65**	-0.09	-0.07	1.00		
eugenol	-0.10	0.31	0.71**	-0.11	0.28	-0.44	-0.27	0.15	1.00	
farnesylacetone	0.29	-0.29	-0.12	0.10	0.10	0.16	0.10	0.65**	-0.29	1.00
octanoic acid	0.11	0.19	-0.21	-0.27	0.51*	-0.12	0.04	0.41	-0.11	0.36
nonanoic acid	0.56**	-0.42	0.39	-0.26	-0.07	-0.27	-0.24	0.01	0.32	0.24

Data used for the Pearson correlation are derived from each of the 20 cultivars as mean from both years. Significant correlation is indicated by asterisks: *p≤0.05 and **p≤0.01. The VOCs which showed no significant correlations with other VOCs were not included in the table

Table S8. Result of principal component analysis (PCA) on the VOCs, instrumental and sensory traits of each of the 20 cultivars as mean from both years showing the scores on the significant principal component (PC)

**Cultivar**	**PC 1**	**PC 2**	**PC 3**	**PC 4**
Goldita	**-4.71**	1.19	1.49	-2.25
Supersweet 100 F_1	**-4.77**	-0.27	2.63	2.89
Resi	-2.19	0.12	**-3.88**	1.71
Bartelly F_1	-2.42	-0.91	0.58	0.46
Benarys Gartenfreude	-3.66	-1.81	-1.88	2.37
Primavera	-1.81	-1.59	0.64	-2.24
Black Cherry	-2.75	-1.13	-1.42	-1.05
SakuraF_1	-2.96	0.32	1.28	0.91
Primabella	-0.23	0.00	-2.98	-1.95
TasteryF_1	0.36	-3.76	2.16	**-5.12**
Annamay F_1	-0.83	-1.08	-0.78	0.88
Amoroso F_1	-0.79	-1.15	0.82	0.37
Campari F_1	1.42	-0.64	0.68	1.15
Auriga	1.53	**7.97**	**4.59**	0.14
Harzfeuer F_1	2.46	-0.09	0.51	1.17
Roterno F_1	**5.33**	-1.21	-1.52	0.84
Lyterno F_1	**5.05**	-1.98	2.15	1.19
Bocati F_1	**5.13**	-0.37	-1.50	-0.78
Cappricia F_1	**5.32**	-0.99	0.56	0.84
Green Zebra	0.53	**7.37**	**-4.15**	-1.54
**Total variance (%)**	23.94	17.26	10.92	7.86

Data used for PCA are derived for each of the 20 cultivars as mean from both years. The percentage variance accounted for by each principal component (PC). Tomato cultivars in each PC, in proportion to the magnitude of their variation value (**bold numeric**), are independent from the tomato cultivars in the other PC. Tomato cultivars in the same PC are related to each other, according to positive and negative variation

Table S9. Loading scores for principal component analysis (PCA) of volatiles of 20 tomato cultivars

**VOCs**	**Principal component (PC)**			
	PC 1	PC 2	PC 3	PC 4
hexanal	0.01	-0.26	-0.47	**-0.53**
mebuOAc	-0.15	0.03	-0.40	0.22
hexenal	**-0.63**	0.25	0.35	-0.06
octanal	0.05	-0.56	**0.56**	0.11
meheptone	-0.11	0.12	-0.38	**0.64**
hexanol	-0.49	-0.04	-0.17	-0.14
hexenol	**-0.60**	-0.05	0.43	0.00
isobut	**0.74**	0.08	-0.05	0.12
meheptenol	**0.69**	-0.32	-0.03	0.18
ethexanol	-0.05	-0.33	**0.61**	-0.49
benzald	0.19	**0.51**	0.30	0.36
linalool	0.28	0.20	0.34	0.33
meheptdione	0.13	-0.05	-0.05	0.21
cyclocit	0.05	0.35	**0.70**	-0.05
mebutacid	0.38	**0.59**	-0.45	0.25
terpineol	-0.10	-0.07	0.04	0.13
geranial	0.37	-0.75	0.13	0.28
citral	-0.53	-0.55	0.00	0.39
deca	-0.29	-0.18	-0.17	0.31
meOSal	0.26	-0.15	0.03	**-0.56**
damasc	0.00	**0.57**	0.33	0.23
gera	-0.21	-0.16	0.28	-0.25
mepropacid	0.04	0.11	**0.75**	-0.31
benzylalc	**-0.59**	0.12	0.29	-0.06
phenylet	**-0.59**	0.00	0.34	0.11
ionone	0.06	0.21	**0.74**	-0.13
eugenol	0.42	-0.30	0.08	-0.48
farnesylac	-0.03	**0.51**	0.42	0.26
octacid	0.11	0.20	0.57	**0.55**
nonacid	0.31	**0.63**	-0.21	-0.17
TSS	**-0.91**	-0.22	0.03	0.12
TA	-0.70	**0.56**	-0.20	0.15
L*	0.30	**0.82**	0.13	-0.22
a*	0.37	-0.53	0.19	0.38
b*	0.32	**0.79**	0.20	-0.14
C*	0.49	**0.67**	0.12	0.01
h	-0.16	**0.72**	-0.09	-0.41
fruit firmness	0.55	-0.56	0.09	-0.19
juiciness	-0.24	**0.55**	-0.46	-0.36
skin firmness	-0.77	0.21	0.16	0.13
sweetness	**-0.92**	-0.17	0.14	-0.08
sourness	-0.60	**0.66**	-0.22	0.05
aroma	**-0.92**	0.22	-0.11	0.07
acceptability	**-0.85**	0.08	0.01	-0.02

Cocktail cultivar (fruit type)	-0.84	-0.37	-0.06	-0.17
Salad cultivar (fruit type)	0.84	0.37	0.06	0.17
**Total variance (%)**	23.94	17.26	10.92	7.86

The percentage variance accounted for by each principal component (PC). The variables in each PC, in proportion to the magnitude of their variation value (**bold numeric**), are independent from the variables in the other PC. Variables in the same PC are related to each other, according to positive and negative variation
